# Symanzik improvement of the gradient flow in lattice gauge theories

**DOI:** 10.1140/epjc/s10052-015-3831-9

**Published:** 2016-01-13

**Authors:** Alberto Ramos, Stefan Sint

**Affiliations:** PH-TH, CERN, 1211 Geneva 23, Switzerland; School of Mathematics, Trinity College, Dublin, Dublin 2, Ireland

## Abstract

We apply the Symanzik improvement programme to the $4+1$-dimensional local re-formulation of the gradient flow in pure *SU*(*N*) lattice gauge theories. We show that the classical nature of the flow equation allows one to eliminate all cutoff effects at $\mathcal O(a^2)$, which originate either from the discretised gradient flow equation or from the gradient flow observable. All the remaining $\mathcal O(a^2)$ effects can be understood in terms of local counterterms at the zero flow-time boundary. We classify these counterterms and provide a complete set as required for on-shell improvement. Compared to the 4-dimensional pure gauge theory only a single additional counterterm is required, which corresponds to a modified initial condition for the flow equation. A consistency test in perturbation theory is passed and allows one to determine all counterterm coefficients to lowest non-trivial order in the coupling.

## Introduction

In recent years the Yang–Mills gradient flow has been established as a very promising new tool to study non-perturbative aspects of strongly coupled gauge theories [1–4]. The gradient flow defines a deterministic mapping from the original gauge field $A_\mu (x)$ to a smoothed gauge field configuration, $B_\mu (t,x)$, at flow time *t*, which is obtained as the solution of the gradient flow equation (see Appendix A for a summary of our conventions),

1 $$\begin{aligned} \partial _t B_\mu (t,x) = \sum _{\nu } D_\nu G_{\nu \mu }(t,x),\quad B_\mu (0,x) = A_\mu (x), \end{aligned}$$

where $D_\mu = \partial _\mu + [B_\mu ,\cdot ]$ denotes the gauge covariant derivative and

2 $$\begin{aligned} G_{\mu \nu }= \partial _\mu B_\nu - \partial _\nu B_\mu + [B_\mu ,B_\nu ], \end{aligned}$$

is the associated field strength tensor. The name relates to the fact that the right hand side of (1) is equal to minus the gradient of the Yang–Mills gauge action. Hence, with increasing flow time *t*, the solution, $B_\mu (t,x)$, is driven towards a minimum of the action and thus approaches a smooth classical field configuration.

There is quite some freedom when translating the gradient flow equation to a Euclidean space-time lattice. A simple possibility is to choose Wilson’s plaquette action, $S_\mathrm{W}$, and to define the lattice gauge field at finite flow time, $V_\mu (t,x)$, as the solution of the Wilson flow equation,

3 $$\begin{aligned} a^{2} [\partial _t V_\mu (t,x)]V_\mu (t,x)^\dagger = -g_0^2\partial _{x,\mu } S_{\text {W}}[V], \end{aligned}$$

where $\partial _{x,\mu }$ denotes the Lie-algebra valued derivative with respect to $V_\mu (t,x)$. It should be noted that similar smoothing operations have long been successfully applied in lattice QCD. For example, the stout link smearing technique of Ref. [5] can be understood as a discretised flow time version of Eq. (3). The essential new element is a theoretical understanding of the renormalisation properties of the Yang–Mills gradient flow. In particular, in [3, 4] it was proved to all orders of perturbation theory that QCD at finite flow time *t* is renormalised once it is renormalised at flow time $t=0$ through the usual renormalisations of the gauge coupling and the quark mass parameters. Furthermore, gauge invariant fields at positive flow time are automatically renormalised and do not mix with other fields of the same or lower dimensions. These properties allow one to define a new class of renormalised gauge invariant observables which can be used to probe the theory in various ways. It also opens new ways to define renormalised composite operators at zero flow time; the study of Ward identities at positive flow times [4, 6, 7] and the applications of the so called “small flow-time expansion” have received much attention recently in this context [8–10].

Many current lattice QCD applications of the gradient flow only involve the simplest possible gauge invariant field, the action density,

4 $$\begin{aligned} E(t,x)= -\frac{1}{2}\sum _{\mu ,\nu } \mathrm{tr}\{ G_{\mu \nu }(t,x)G_{\mu \nu }(t,x)\}. \end{aligned}$$

As initially proposed in [2], the expectation value $\langle E(t,x)\rangle$ can be used for a non-perturbative definition of either a reference scale or a coupling constant. This has proven very attractive: in large volume simulations it leads to the most precise determination of a reference scale (for a recent review cf. [11]). On the other hand, when considered in a finite space-time volume the scale evolution of the corresponding coupling [12–16] can be traced with high statistical precision (see [17] for a recent review).

Notwithstanding these nice properties a major practical problem consists in the relatively large cutoff effects which have been observed in several applications (cf. [17] and references therein). On general grounds, the leading effects are expected to be of order $a^2$. Their size depends on the detailed choices made when translating the flow equation (1) to the lattice, but also on the discretisation of the observable and on the lattice action. Alternative flow equations have been tried, e.g. in Ref. [18] where the Wilson action was replaced by the tree-level improved Lüscher–Weisz action, $S_\mathrm{LW}$ [19, 20]. For some attempts to reduce cutoff effects in the particular observable $\langle E(t,x)\rangle$ cf. Refs. [21, 22]. Here we would like to proceed more systematically by applying the Symanzik procedure [19, 23] to the $4+1$-dimensional local formulation of the theory [3, 24]. This will lead us to a particular choice for the lattice flow equation, referred to as the “Zeuthen flow” and defined by

5 $$\begin{aligned} a^2(\partial _t V_\mu (t,x)) V_\mu (t,x)^\dagger= & {} -g_0^2 \left( 1 + \frac{a^2}{12}\nabla _\mu ^*\nabla _\mu ^{} \right) \nonumber \\&\times \partial _{x,\mu } S_\mathrm{LW}[V], \end{aligned}$$

with the initial condition $V_\mu (0,x) = U_\mu (x)$. Here $\nabla _\mu ^{}$ and $\nabla _\mu ^*$ are the lattice forward and backward covariant derivatives, respectively. We will show that the integration of the Zeuthen flow equation does not generate any cutoff effects at $\mathcal O(a^2$). If combined with classical $\mathcal O(a^2)$ improvement of the observable all $\mathcal O(a^2)$ effects are eliminated apart from those corresponding to local counterterms in the action at zero flow time. We will give a complete list of such counterterms and test our framework to lowest non-trivial order in perturbation theory.

The paper is organised as follows: in Sect. 2 we recall the definition of the $4+1$-dimensional local theory, with flow time as the added dimension. In Sect. 3 we discuss the general Symanzik procedure and the simplifications due to the special properties of this theory. We present the classical *a*-expansion of both the flow action and the gradient flow observable *E*(*t*, *x*), as part of the simplified Symanzik procedure, and carry out the standard Symanzik analysis for the $\mathcal O(a^2)$ counterterms at the $t=0$ boundary. Section 4 presents a number of perturbative tests of the $\mathcal O(a^2)$ improved theory, and Sect. 5 our conclusions. We have included three appendices regarding our notations and conventions (Appendix A), some technical details pertaining to the classical *a*-expansion (Appendix B), and some explicit expressions used in Sect. 4 (Appendix C), respectively.

## Lattice gauge theory in $4+1$ dimensions

The gradient flow equation can be viewed as a way to define a particular class of observables, i.e. fields which are functionals of the fundamental gauge field $U_\mu (x)$. The flow time thus appears as an additional parameter which measures the range in space-time over which the fundamental gauge field enters into an observable defined in terms of the flowed gauge field $V_\mu (t,x)$. The flow time *t* has dimension length squared and the “smearing radius” $r_t=\sqrt{8t}$ is usually taken as the corresponding length scale.^1^ Thus, gradient flow observables are non-local objects from the perspective of the 4-dimensional gauge theory and their properties under renormalisation are difficult to assess. Moreover, the non-locality prevents a straightforward application of the Symanzik expansion, which is our main theoretical tool for understanding the cutoff dependence of the theory. For this purpose, it is therefore highly beneficial to follow [4] and view the theory from a $4+1$-dimensional perspective, with flow time as the added dimension. In this re-formulation locality is restored in the $4+1$-dimensional sense, and dimensional counting can be applied to classify counterterms to the action and observables.

We start with the formulation of the lattice set-up, including the introduction of a flow-time lattice. The latter should be regarded as an intermediate regularisation which helps to resolve certain technical issues [4]. While none of this is original it serves for later reference and to fix our notation.

### The 4-dimensional lattice action

**Fig. 1:**
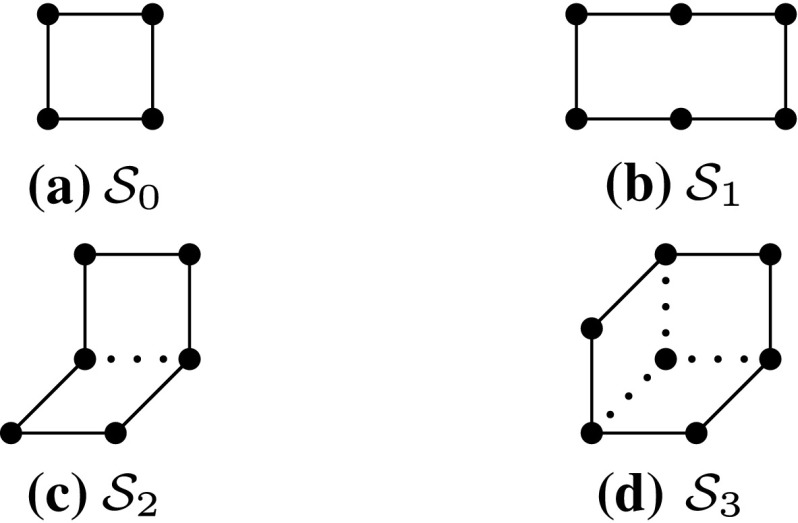
Wilson loops of type $\mathcal S_0, \mathcal S_1, \mathcal S_2$ and $\mathcal S_3$

On-shell $\mathcal O(a^2)$ improvement of the 4-dimensional gauge theory can be achieved by introducing, besides the 4-link plaquette action, further 6-link Wilson loops with appropriately chosen coefficients [19]. We will consider a general class of lattice gauge actions parameterised by the coefficients $c_i (i=0,1,2,3)$, defined by,

6 $$\begin{aligned} S_{\text {g}}[U,\{c_i\}] = \frac{1}{g_0^2} \sum _{i=0}^3 c_i \sum _{\mathcal W\in \mathcal S_i} \mathrm{Tr}(1 - U(\mathcal C)), \end{aligned}$$

where the second sum extends over all oriented Wilson loops of type $\mathcal S_i$. As illustrated in Fig. 1, these Wilson loops are the usual plaquettes, $\mathcal S_0$, the $2\times 1$ planar loops or “rectangles”, $\mathcal S_1$, the bent rectangles or “chairs”, $\mathcal S_2$, and finally the “parallelograms”, $\mathcal S_3$.

It is conventional to normalise the action by requiring

7 $$\begin{aligned} c_0+ 8c_1+16 c_2 +8 c_3 =1, \end{aligned}$$

such that the standard continuum Yang–Mills action is obtained in the classical continuum limit, with any choice of the three free parameters.^2^ Popular choices are the Wilson plaquette (*W*) action ($c_0=1$, $c_{1,2,3}=0$) and the tree-level improved Lüscher–Weisz (LW) action ($c_0=5/3$, $c_1=-1/12$, $c_{2,3}=0$).

### $4+1$-dimensional set-up

Given the 4-dimensional action, the flow equation is now incorporated in the action as a constraint, by introducing the Lagrange multiplier field $L_\mu (t,x)$, which is hermitian and such that $\imath L_\mu (t,x)$ is Lie-algebra valued. The $4+1$-dimensional action of this theory then takes the form

8 $$\begin{aligned} S[V,L]= & {} S_{\text {g}}[U,\{c_i\}] \nonumber \\&- 2a^4\int _0^\infty \mathrm{d}t\, \sum _{x,\mu } \mathrm{tr}\{L_\mu (t,x) F_\mu (t,x)\}, \end{aligned}$$

where the boundary condition,

9 $$\begin{aligned} V_\mu (0,x) = U_\mu (x), \end{aligned}$$

is assumed and

10 $$\begin{aligned} \begin{array}{ll} F_\mu (t,x) =&{} a^{-1} (\partial _t V_\mu (t,x))V_\mu (t,x)^\dagger \\ &{}+\, a^{-3} g_0^2 \partial _{x,\mu } S_{\text {g}}[V], \end{array} \end{aligned}$$

is a shorthand notation which allows one to write the lattice gradient flow equation in the form $F_\mu (t,x)=0$. The action $S_{\text {g}}[V]$ is some 4-dimensional lattice gauge action for the flowed field $V_\mu (t,x)$, the Wilson action being the simplest choice [cf. Eq. (3)]. In any case it is unrelated to the gauge action $S_{\text {g}}[U]$ in (8). How to best define $F_\mu (t,x)$ is at the core of this work and will be discussed in the next section.

Given the action for the $4+1$-dimensional half space $t\ge 0$, expectation values of composite fields *O*[*V*, *L*] are defined as usual,

11 $$\begin{aligned} \langle O\rangle = \mathcal{Z}^{-1}\int D[V] D[L] O[V,L] \exp \left( - S[V,L]\right) , \end{aligned}$$

with the condition $\langle 1 \rangle =1$. A few remarks are in order: first, the integration over the gauge field $V_\mu (t,x)$ includes the integration over its boundary values at $t=0$ i.e. the standard 4-dimensional gauge field $U_\mu (x)$. Hence, for observables which only depend on $U_\mu$, the functional integrals over $V_\mu \vert _{t>0}$ and $L_\mu$ cancel between numerator and denominator, reproducing the standard expectation value of the 4-dimensional theory. To see this more explicitly it is convenient to pass to a flow-time lattice with spacing $\varepsilon$ and lattice points $t=n\varepsilon$ [4],

12 $$\begin{aligned} \int _0^\infty \mathrm{d}t\, a^4\sum _{x,\mu }&\mathrm{tr}\{L_\mu (t,x) F_\mu (t,x)\} \nonumber \\ \varepsilon \sum _{n\ge 0} a^4\sum _{x,\mu }\rightarrow&\mathrm{tr}\{L_\mu (t,x) F_\mu (t,x;\varepsilon )\}, \end{aligned}$$

where we have assumed the discretisation,

13 $$\begin{aligned} \begin{array}{ll} a\varepsilon F_\mu (t,x;\varepsilon ) =&{} V_\mu (t+\varepsilon ,x) V_\mu (t,x)^\dagger \\ &{}- \exp \left( -g_0^2\frac{\varepsilon }{a^2}\partial _{x,\mu } S_{\text {g}}[V]\right) , \end{array} \end{aligned}$$

with the correct $\varepsilon \rightarrow 0$ limit. Inserting this representation of the action into the functional integral, the integration over the fields $L_\mu (t,x)$ produces a string of $\delta$-functions^3^

14 $$\begin{aligned} \prod _{x,\mu }\prod _{n=0}^\infty \delta [F_\mu (n\varepsilon ,x;\varepsilon )]. \end{aligned}$$

These can be eliminated one by one, by integrating over $V_\mu (n\varepsilon ,x)$ for strictly positive *n*, leaving the unconstrained $n=0$ integration over the fundamental gauge field intact, as expected.

### Gauge symmetry

By construction, the $4+1$-dimensional action is gauge invariant under *t*-independent gauge transformations,

15 $$\begin{aligned} V_\mu (t,x) \rightarrow \Lambda (x) V_\mu (t,x) \Lambda (x+a\hat{\mu })^\dagger , \end{aligned}$$

where $\Lambda (x)$ is an *SU*(*N*)-valued gauge function. This leads to the transformation,

16 $$\begin{aligned} F_\mu (t,x) \rightarrow \Lambda (x) F_\mu (t,x) \Lambda (x)^\dagger , \end{aligned}$$

so that gauge invariance of the action is guaranteed provided that

17 $$\begin{aligned} L_\mu (t,x) \rightarrow \Lambda (x) L_\mu (t,x) \Lambda (x)^\dagger , \end{aligned}$$

i.e. $L_\mu (t,x)$ must be in the adjoint representation of the gauge group. The integration measure is invariant under such a change of variables, so that the gauge symmetry of the 4-dimensional boundary theory is inherited by the bulk theory.

It is occasionally useful to generalise the gauge symmetry to the flow-time coordinate *t*, i.e. admit flow-time dependent gauge functions $\Lambda (t,x)$. In the continuum theory this amounts to replacing $t=x_4$, $\partial _t \rightarrow D_4=\partial _4 + [B_4,\cdot ]$ and $\partial _t B_\mu (t,x) \rightarrow G_{4\mu }(x_4,x)$ [1]. In the presence of the lattice cutoff (but continuous $t=x_4$) we define the covariant $x_4$-derivative by

18 $$\begin{aligned} \begin{array}{ll} \nabla _4 V_\mu (\tilde{x}) =&{} \partial _4 V_\mu (\tilde{x}) + B_4(\tilde{x}) V_\mu (\tilde{x}) \\ &{}-V_\mu (\tilde{x})B_4(\tilde{x}+a\hat{\mu }), \end{array} \end{aligned}$$

where $\tilde{x} = (x_4,x)$. This, together with the transformation under an $x_4$-dependent gauge transformation

19 $$\begin{aligned} B_4(\tilde{x}) \rightarrow \Lambda (\tilde{x}) B_4(\tilde{x}) \Lambda (\tilde{x})^\dagger + \Lambda (\tilde{x})\partial _4 \Lambda (\tilde{x})^\dagger , \end{aligned}$$

leads to the left hand side of the covariant flow equation transforming as

20 $$\begin{aligned}{}[\nabla _4 V_\mu (\tilde{x})] V_\mu (\tilde{x})^\dagger \rightarrow \Lambda (\tilde{x})[\nabla _4 V_\mu (\tilde{x})] V_\mu (\tilde{x})^\dagger \Lambda (\tilde{x})^\dagger . \end{aligned}$$

Rendering the *t*-derivative covariant in the definition of $F_\mu$ (Eq. 10), one then obtains,

21 $$\begin{aligned} F_\mu (\tilde{x}) \rightarrow \Lambda (\tilde{x}) F_\mu (\tilde{x}) \Lambda (\tilde{x})^\dagger , \end{aligned}$$

so that $4+1$-dimensional gauge invariance is established, provided that $L_\mu (\tilde{x})$ transforms just like $F_\mu (\tilde{x})$. Discretising the flow-time coordinate is also straightforward, one just needs to elevate the fourth component of the gauge field $B_4$ to a link field $V_4$, with corresponding changes in the covariant derivative and gauge transformation behaviour.

Finally we note that the Yang–Mills flow equation in the continuum can be written as

22 $$\begin{aligned} G_{4\mu } = \sum _{\nu =0}^3 D_\nu G_{\nu \mu }, \end{aligned}$$

which shows that the $4+1$-dimensional theory, while exactly gauge invariant, does not enjoy any generalised Lorentz symmetry. This is of course already clear from the dimensions, in particular, $\partial _t$ and thus $B_4$ must have mass dimension 2, in contrast to the usual derivatives and gauge fields in 4 dimensions.

## Symanzik improvement to $\mathcal O(a^2)$

### Generalities

The re-formulation of gradient flow observables in terms of a local $4+1$-dimensional lattice gauge theory creates the standard situation to which Symanzik’s effective theory [23] can be applied in the usual way. We start with Symanzik’s effective action which is given as an expansion in powers of $a^2$,

23 $$\begin{aligned} \begin{array}{ll} S_\text {eff}[B,L] =&{} S_0^{\text {cont}}[B,L] + a^2 S_{2,\mathrm{fl}}[B,L] \\ &{}+\, a^2 S_{2,b}[B,L] + O(a^4). \end{array} \end{aligned}$$

One might worry about odd powers of *a* arising in a $4+1$-dimensional theory. However, as we will show in detail in Appendix B, gauge invariance, reflection symmetries and the fact that the flow-time parameter *t* has mass dimension $-2$ imply that non-trivial counterterms to the action must be even dimensional. In Eq. (23) we have separated the effective action of the flow in the $4+1$-dimensional volume, $S_{2,\mathrm{fl}}$, from the action $S_{2,b}$ with support restricted to the 4-dimensional boundary at $t=0$. Both parts will be discussed in turn below. Besides the effective action, also local observables are described by an effective continuum field, again expanded in powers of $a^2$. For a generic local observable $\mathcal{O}$ we write

24 $$\begin{aligned} \mathcal{O}_{\text {eff}} = \mathcal{O}_0 + a^2 \mathcal{O}_2 + \mathcal O(a^4). \end{aligned}$$

To $\mathcal O(a^2)$ the Symanzik expansion of lattice expectation values then takes the form,

25 $$\begin{aligned} \begin{array}{ll} \langle \mathcal{O} \rangle ^\text {lat} =&{} \langle \mathcal{O}_0 \rangle + a^2 \langle \mathcal{O}_2 \rangle \\ &{}-\, a^2 \langle \mathcal{O}_0 S_{2,\mathrm{fl}} \rangle _c - a^2 \langle \mathcal{O}_0 S_{2,b}\rangle _c +\mathcal O(a^4). \end{array} \end{aligned}$$

Here, the expectation values on the RHS are defined in the continuum theory with respect to the continuum action $S_0^{\text {cont}}$, and the notation $\langle \cdot \rangle _c$ serves as a reminder that only the connected part contributes to the correlation functions with counterterm insertions, for instance

26 $$\begin{aligned} \langle \mathcal{O}_0 S_{2,\mathrm{fl}} \rangle _c = \langle \mathcal{O}_0 S_{2,\mathrm{fl}} \rangle - \langle \mathcal{O}_0 \rangle \langle S_{2,\mathrm{fl}} \rangle . \end{aligned}$$

As the next step in the Symanzik procedure one determines a basis of counterterms both for the action and the observables of interest. In the case of the action these take the form

27 $$\begin{aligned} S_{2,\mathrm{fl}}[B,L]= & {} \int _0^\infty \mathrm{d}t \int \mathrm{d}^4x\,\sum _{i=1}^{n_\mathrm{fl}} Q_i(t,x), \end{aligned}$$

28 $$\begin{aligned} S_{2,b}[B,L]= & {} \int \mathrm{d}^4x\,\sum _{i=1}^{n_b} O_i(x), \end{aligned}$$

where the fields $Q_i(t,x)$ are gauge invariant polynomials in the fundamental fields $B_\mu (t,x)$, $L_\mu (t,x)$ and their (space-time and/or flow-time) derivatives, and the $O_i(x)$ are similarly constructed, but evaluated at $t=0$. Since $a^2 S_{2,\mathrm{fl}}$ must be dimensionless, the fields $Q_i$ must have mass dimension 8 and otherwise share all the symmetries with the lattice theory. The fields $O_i$ are dimension 6 fields, localised at the $t=0$ boundary. One of the important outcomes of the Symanzik analysis are the numbers $n_\mathrm{fl}$ and $n_b$ of basis elements, where fields differing by total (space-time) derivative terms are considered equivalent. Furthermore, restricting to on-shell improvement the field equations for $L_\mu$, $B_\mu$ and $A_\mu$ can be used to simplify the basis. Given a basis of counterterms the final step of Symanzik’s procedure consists in adding lattice representatives of these operators to the lattice action, such that, with appropriately chosen coefficients, the terms $S_{2,\mathrm{fl}}$ and $S_{2,b}$ are eliminated in Symanzik’s effective action for the improved lattice action.

A similar analysis then needs to be carried out for each observable $\mathcal{O}$ of interest, i.e. $\mathcal{O}_2$ in Eq. (24) is given as a linear combination of local fields of mass dimension $\text {dim}(\mathcal{O}_0)+2$, which share all the lattice symmetries with $\mathcal{O}$. While this procedure applies to any observables, we will here focus on gradient flow observables, i.e. gauge invariant composite fields with support at strictly positive flow times.

If the full Symanzik procedure as outlined above were really necessary, $\mathcal O(a^2)$ improvement would probably remain an academic curiosity. In particular, a rather long list of dimension 8 counterterms for $S_{2,\mathrm{fl}}$ could be written down, with little hope for practical relevance, so that one might be tempted to give up on systematic $\mathcal O(a^2)$ improvement.

Before proceeding along these lines, however, it is advisable to have a closer look at this particular theory. As shown by Lüscher and Weisz, the theory is perturbatively renormalizable to all orders in the 4-dimensional gauge coupling *g* [3]. More precisely, if one restricts attention to gauge invariant observables, one just needs to renormalise the gauge coupling in the usual way, and also the quark masses if the boundary theory is generalised to QCD.^4^ Moreover, any composite fields defined at finite flow time are automatically renormalised and do not mix with any other fields of the same or lower canonical dimension. The action density (4) is a typical example: its renormalisation at flow time $t=0$ requires the subtraction of both a quartic and a logarithmic divergence. None of this is required at finite *t*. It is instructive to consider leading order perturbation theory to get a basic understanding of the mechanism at work. Effectively, at finite flow time *t*, integrals over the loop-momentum *p* are cut off by an exponential suppression factor $\propto \exp (-2tp^2)$ in the integrand. This renders most momentum integrals finite, so that one is only left with those divergences which are cancelled by the standard counterterms in the boundary theory.

Hence the $4+1$-dimensional theory enjoys rather special properties. In particular, the field $L_\mu$ plays the rôle of a Lagrange multiplier field which enforces the gradient flow equation as a constraint. The smoothening properties of this equation are related to the fact that perturbation theory only generates tree diagrams for the correlation functions of gradient flow observables [3]. The Symanzik expansion is then very much simplified as we expect the following to hold:

- The absence of bulk loop diagrams in the perturbative expansion of gradient flow observables implies that classical improvement of the flow action cancels the $\mathcal O(a^2)$ effects *exactly*, i.e. without any corrections.
- By the same argument, non-perturbative $\mathcal O(a^2)$ improvement of composite operators at positive flow time can be achieved by choosing discretisations that do not generate $\mathcal O(a^2)$ effects when expanded classically.
- The only $\mathcal O(a^2)$ counterterms which receive genuine quantum corrections are the ones living in the 4-dimensional boundary at $t=0$. The full Symanzik procedure outlined above thus needs to be applied only to the $t=0$ boundary part, $S_{2,b}$, of the Symanzik action, and of course to any observable which is at least in part localised at the $t=0$ boundary.

In the following we first remind the reader of the classical *a*-expansion and then address these points in the subsequent subsections one at a time.

### The classical *a*-expansion

According to the preceding discussion the counterterms appearing in $S_{2,\mathrm{fl}}$ and in $\mathcal{O}_2$ for gradient flow observables are completely determined by classically expanding the lattice action in the $4+1$-dimensional volume and the observables under consideration to order $a^2$. The classical expansion assumes that the lattice approximates an underlying continuum space-time manifold on which a smooth continuum gauge field, $B_\mu (t,x)$, is defined. The lattice gauge field, $V_\mu (t,x)$, is then related to the continuum gauge field by parallel transport along the lattice links. Parameterising the path along the lattice link from $x+a\hat{\mu }$ to *x* by $z(u) = x + (1-u)a\hat{\mu }$ (with parameter $u\in [0,1]$), the precise relation is obtained by iteratively solving the differential equation,29The solution, $v(u=1)\equiv V_\mu (t,x)$, can be concisely written in terms of a path-ordered exponential,30While it is straightforward to carry out the expansion around $a=0$, in practice, even a simple gauge invariant quantity like the trace of the plaquette contains four link variables which need to be expanded and combined to fourth order in *a* to obtain the leading non-trivial term. It is therefore highly advisable to perform the expansion efficiently (cf. e.g. [19, 25]). We here follow Lüscher and Weisz [19], who, for fixed indices $\mu$ and $\nu$, proposed to work in the following gauge:

31 $$\begin{aligned} B_\mu (t,x) = 0 \quad \text { for all } x; \quad B_\nu (t,x) = 0 \quad \text { if }x_\mu =0. \end{aligned}$$

As a result, the expansion around $x=0$ is very much simplified. For example, the plaquette field,

32 $$\begin{aligned} P_{\mu \nu }(t,x) = V_\mu (t,x)V_\nu (t,x+a\hat{\mu })V_\mu (t,x+a\hat{\nu })^\dagger V_\nu (t,x)^\dagger , \end{aligned}$$

is reduced to a single link,

33 $$\begin{aligned} P_{\mu \nu }(t,0)= & {} V_\nu (t,a\hat{\mu }) \nonumber \\= & {} \mathcal{P} \exp \left\{ a \int _0^1 \mathrm{d}u B_\nu \left( t,a\hat{\mu }+ (1-u)a\hat{\nu }\right) \right\} .\nonumber \\ \end{aligned}$$

Recalling the definition of the path-ordered exponential (30) one needs the expansion of the *B*-field around $a=0$,

$$\begin{aligned} \begin{array}{ll} &{}aB_\nu (t,a\hat{\mu }+ \kappa a\hat{\nu })= a^2\partial _\mu B_\nu (t,0) \\ &{}\quad +\, \frac{1}{2} a^3\{\partial _\mu ^2+ 2\kappa \partial _\mu \partial _\nu \} B_\nu (t,0)\\ &{}\quad +\, \frac{1}{6} a^4\{ \partial _\mu ^3+3\kappa \partial _\mu ^2\partial _\nu ^{} + 3\kappa ^2\partial _\mu ^{}\partial _\nu ^2\} B_\nu (t,0)\\ &{}\quad +\, \frac{1}{24} a^5 \{\partial _\mu ^4 + 4\kappa \partial _\mu ^3\partial _\nu ^{} +6\kappa ^2\partial _\mu ^2\partial _\nu ^2 +\, 4 \kappa ^3\partial _\mu ^{}\partial _\nu ^3\}B_\nu (t,0) \\ &{}\quad + \cdots , \end{array} \end{aligned}$$

where $\kappa$ is a constant and neglected terms are of order $a^6$. Following [19] the gauge covariant expressions can be *unambiguously* restored, with the result

$$\begin{aligned} \begin{array}{ll} &{}aB_\nu (t,a\hat{\mu }+ \kappa a\hat{\nu }) = a^2 G_{\mu \nu }(t,0) \\ &{}\quad +\, \frac{1}{2} a^3\{D_\mu + 2\kappa D_\nu \} G_{\mu \nu }(t,0) \\ &{}\quad +\, \frac{1}{6} a^4\{ D_\mu ^2 + 3\kappa D_\nu D_\mu + 3\kappa ^2 D_\nu ^2\} G_{\mu \nu }(t,0)\\ &{}\quad +\, \frac{1}{24} a^5 \{D_\mu ^3 + 4\kappa D_\nu ^{} D_\mu ^2 + 6\kappa ^2 D_\nu ^2 D_\mu ^{} + 4\kappa ^3 D_\nu ^3\}G_{\mu \nu }(t,0) \\ &{}\quad +\, \cdots \end{array} \end{aligned}$$

Inserting into the path-ordered exponential with appropriate replacements for $\kappa$, we thus obtain the gauge covariant expansion for the plaquette field,34which holds for any argument (*t*, *x*). Similar expressions can be derived for the other three plaquettes in the $\mu -\nu$ plane:

35 $$\begin{aligned} Q_{\mu \nu }(t,x)= & {} V_\nu (t,x-a\hat{\nu })^\dagger V_\mu (t,x-a\hat{\nu }) \nonumber \\&\times V_\nu (t,x+a\hat{\mu }-a\hat{\nu })V_\mu (t,x)^\dagger , \end{aligned}$$

36 $$\begin{aligned} R_{\mu \nu }(t,x)= & {} V_\mu (t,x-a\hat{\mu })^\dagger V_\nu (t,x-a\hat{\mu }-a\hat{\nu })^\dagger \nonumber \\&\times V_\mu (t,x-a\hat{\mu }-a\hat{\nu }) V_\nu (t,x-a\hat{\nu }), \end{aligned}$$

37 $$\begin{aligned} S_{\mu \nu }(t,x)= & {} V_\nu (t,x)V_\mu (t,x-a\hat{\mu }+a\hat{\nu })^\dagger \nonumber \\&\times V_\nu (t,x-a\hat{\mu })^\dagger V_\mu (t,x-a\hat{\mu }), \end{aligned}$$

and the next few orders can be obtained with moderate additional effort.

### Determination of $S_{2,\mathrm{fl}}$

To find the bulk counterterm action $S_{2,\mathrm{fl}}$ we simply need to apply the classical expansion to the bulk action in Eq. (8). This essentially amounts to the *a*-expansion of the gradient flow equation, i.e. $F_\mu (t,x)$ in Eq. (10). For the first term we find, in the Lüscher–Weisz gauge (31),

38 $$\begin{aligned} a^{-1} [\partial _tV_\mu (t,0)]V_\mu (t,0)^\dagger = \int _0^1 \mathrm{d}u\, \partial _t B_\mu (t,(1-u)a\hat{\mu }), \end{aligned}$$

as all other terms are proportional to $B_\mu (t,0)=0$. The Taylor expansion can easily be performed to all orders in *a* with the result

39 $$\begin{aligned} \int _0^1 \mathrm{d}u\, \partial _t B_\mu (t,(1-u)a\hat{\mu }) = \sum _{n=0}^\infty \frac{a^n}{(n+1)!}\partial _\mu ^n\partial _tB_\mu (t,0). \end{aligned}$$

We therefore expect that the correct gauge covariant expression at any lattice point *x* must read

40 $$\begin{aligned} a^{-1} [\partial _tV_\mu (t,x)]V_\mu (t,x)^\dagger= & {} \partial _tB_\mu (t,x) \nonumber \\&+ \sum _{n=1}^\infty \frac{a^n}{(n+1)!} D_\mu ^n\partial _tB_\mu (t,x). \nonumber \\ \end{aligned}$$

At this point one may wonder whether the gauge covariant expression really follows unambiguously from the gauge fixed expansion, in particular, whether the *t*-derivative always has to be to the right of the covariant $\mu$-derivatives. That this is indeed correct can be established by using the $4+1$-dimensional gauge symmetry (cf. Sect. 2), which implies that the *a*-expansion of this term must be given as covariant derivatives acting on $G_{4\mu }$.

Turning to the second term of (10), i.e. the gradient force term, we choose a quite general lattice gauge action parameterised by $c_{0,1,2}$, which includes all four- and six-link Wilson loops (plaquettes, rectangles, chairs) except the parallelograms. We decompose the action as follows:

41 $$\begin{aligned} S_{\text {g}}[V;c_0,c_1,c_2] = c_0 S_{\text {g,pl}}[V] + c_1 S_{\text {g,re}}[V] + c_2 S_{\text {g,ch}}[V]. \end{aligned}$$

We first express the gradient force in terms of plaquettes and their covariant derivatives. For the plaquette action we then find

42 $$\begin{aligned} g_0^2\partial _{x,\mu } S_{\text {g,pl}}[V] = \sum _\nu \left( P_{\mu \nu }(t,x)+Q_{\mu \nu }(t,x)^\dagger \right) _\mathrm{AH}, \end{aligned}$$

where we have introduced the projection on the traceless antihermitian part, i.e. for an $N\times N$ matrix *M* in colour space we define

43 $$\begin{aligned} \left( M\right) _\mathrm{AH} = {\frac{1}{2} (M-M^\dagger )}-{\frac{1}{2N} {\text{ tr }}(M-M^\dagger )} \end{aligned}$$

For the rectangle action we find

$$\begin{aligned}&g_0^2\partial _{x,\mu } S_{\text {g,re}}[V] = \sum _\nu \Bigg ( 2 P_{\mu \nu }(t,x)P_{\mu \nu }(t,x) \nonumber \\&\quad \,-2 Q_{\mu \nu }(t,x)Q_{\mu \nu }(t,x) \nonumber \\&\quad +\, P_{\mu \nu }(t,x)S_{\mu \nu }(t,x) - R_{\mu \nu }(t,x)Q_{\mu \nu }(t,x) \nonumber \\&\quad +\, (a\nabla _\mu P_{\mu \nu }(t,x)) P_{\mu \nu }(t,x) - Q_{\mu \nu }(t,x)a\nabla _\mu Q_{\mu \nu }(t,x) \nonumber \\&\quad +\, (a\nabla _\nu ^*Q_{\mu \nu }(t,x)) Q_{\mu \nu }(t,x) + P_{\mu \nu }(t,x)a\nabla _\nu P_{\mu \nu }(t,x)\Bigg )_\mathrm{AH}, \end{aligned}$$

and a similar but slightly more complicated expression is obtained for the chairs. Expanding each term to order $a^2$ and recombining them we get

$$\begin{aligned} \begin{array}{ll} &{}g_0^2\partial _{x,\mu } S_{\text {g}} = a^3\sum _{\nu } \Biggl \{ \left( c_0+8c_1+16c_2\right) \left( D_\nu G_{\nu \mu } + \frac{a}{2} D_\mu D_\nu G_{\nu \mu }\right) \\ &{}\quad +\, a^2\Biggl [\frac{1}{12}\left( c_0+20c_1+4c_2\right) \left( D_\nu ^3+2D_\nu D_\mu ^2\right) +(c_2-c_1)D_\mu ^2 D_\nu \\ &{}\quad +\, c_2\sum _{\rho }\left( 3 D_\rho ^2 D_\nu - 4D_\rho D_\nu D_\rho + 2 D_\nu D_\rho ^2\right) \Biggr ] G_{\nu \mu }\Biggr \} + \mathcal O(a^6), \end{array} \end{aligned}$$

where the arguments (*t*, *x*) on the RHS have been omitted. Collecting all results we define the expansion coefficients

44 $$\begin{aligned} F_\mu (t,x) = \sum _{n=0}^{\infty } a^n F_\mu ^{(n)}(t,x), \end{aligned}$$

where the leading term defines the continuum limit,

45 $$\begin{aligned} F_\mu ^{(0)}(t,x) = \partial _t B_\mu (t,x) - (c_0+8c_1+16c_2) \sum _\nu D_\nu G_{\nu \mu }(t,x). \end{aligned}$$

Hence the correct normalisation to reproduce the Yang–Mills gradient flow equation (1) is $c_0+8c_1+16c_2=1$, which we use to eliminate $c_0$ in the higher order terms:

$$\begin{aligned} F_\mu ^{(1)}= & {} \frac{1}{2} D_\mu (\partial _t B_\mu - \sum _\nu D_\nu G_{\nu \mu }),\nonumber \\ F_\mu ^{(2)} \!= & {} \! \frac{1}{6} D_\mu ^2 \partial _t B_\mu \!-\! \left( \frac{1}{12} \!+\! c_1\!-\!c_2\right) \sum _{\nu }(2 D_\nu ^{}D_\mu ^2+D_\nu ^3) G_{\nu \mu } \nonumber \\&+ \sum _{\nu }\Bigg [(c_1-c_2) D_\mu ^2D_\nu ^{} \nonumber \\&-c_2\sum _{\rho }(3D_\rho ^2D_\nu ^{}-4D_\rho ^{} D_\nu ^{} D_\rho ^{} +2D_\nu ^{}D_\rho ^2)\Bigg ]G_{\nu \mu }. \end{aligned}$$

Before proceeding we remark on the presence of odd powers of *a* in the expansion, which seems at odds with our expectation that only even powers of *a* occur in this theory. The resolution of this apparent contradiction lies in the fact that the lattice fields $F_\mu (t,x)$ and $L_\mu (t,x)$ should be defined on the lattice link connecting *x* and $x+a\hat{\mu }$, rather than at the lattice site *x*. In Appendix B we demonstrate how the covariant re-expansion about the midpoint of the link, $\tilde{x} = x + \frac{1}{2} a\hat{\mu }$, eliminates such terms. While this problem will not affect our discussion of the $\mathcal O(a^2)$ counterterms, it clarifies that the correction terms are indeed of order $a^4$.

We now proceed and work out the simplifications due to the field equations for $B_\mu (t,x)$ and $L_\mu (t,x)$. Varying the continuum action with respect to $L_\mu$ one obtains the Yang–Mills flow equation (1), whereas the variation with respect to $B_\mu (t,x)$ yields

46 $$\begin{aligned} \partial _t L_\mu = \sum _\nu \left( -D_\mu D_\nu L_\nu - D_\nu ^2 L_\mu + 2 D_\nu D_\mu L_\nu \right) \end{aligned}$$

Using the flow equation eliminates the *O*(*a*) term $F_\mu ^{(1)}$, and this is the reason why the $\mathcal O(a^2)$ terms remain unaffected by the symmetrisation about the midpoint $\tilde{x}$, once the field equations are taken into account. From the continuum flow equation we derive

47 $$\begin{aligned} \partial _t \sum _{\nu }D_\nu G_{\nu \mu } = \sum _{\nu ,\rho }(3D_\rho ^2D_\nu ^{}-4D_\rho ^{} D_\nu ^{} D_\rho ^{} +2 D_\nu ^{}D_\rho ^2)G_{\nu \mu }. \end{aligned}$$

This allows one to rewrite the $\mathcal O(a^2)$ term as follows:

$$\begin{aligned} F_\mu ^{(2)}(t,x)= & {} \sum _\nu \Biggl \{ -\left( \frac{1}{12} + c_1-c_2\right) (2 D_\nu ^{}D_\mu ^2+D_\nu ^3) \\&+\left( \frac{1}{6} + c_1-c_2\right) D_\mu ^2 D_\nu ^{} -c_2\partial _t D_\nu ^{}\Biggr \}G_{\nu \mu }(t,x). \end{aligned}$$

From the corresponding $\mathcal O(a^2)$ flow action,

$$\begin{aligned} S_{2,\mathrm{fl}}[B,L] = -2\int _0^\infty \mathrm{d}t \int \mathrm{d}^4x \sum _{\mu }\mathrm{tr}\{ L_\mu (t,x) F_\mu ^{(2)}(t,x)\}, \end{aligned}$$

one may now directly read off the counterterm structures $Q_i$ that correspond with a given choice of the coefficients $c_{1,2}$. Unfortunately, there does not seem to be a choice such that $S_{2,\mathrm{fl}}$ vanishes. We also attempted to use Eq. (46) as follows: considering the term

48 $$\begin{aligned} 2 c_2 \int _0^\infty \mathrm{d}t \int \mathrm{d}^4x\sum _{\mu ,\nu } \mathrm{tr}\{ L_\mu (t,x) \partial _t D_\nu ^{}G_{\nu \mu }(t,x) \}, \end{aligned}$$

one may perform an integration by parts with respect to *t*. This generates a surface term at $t=0$,

49 $$\begin{aligned} - 2 c_2 \int \mathrm{d}^4x \sum _{\mu ,\nu } \left. \mathrm{tr}\{ L_\mu (t,x) D_\nu ^{}G_{\nu \mu }(t,x) \}\right| _{t=0}, \end{aligned}$$

which re-defines a coefficient of the counterterms entering $S_{2,b}$ (cf. Sect. 3.5). Equation (46) then leads to space-time derivatives acting on $L_\mu$, which can be integrated by parts (no surface terms are generated here) to redefine $F_\mu ^{(2)}$. Unfortunately, this does not yield a solution with $S_{2,\mathrm{fl}} = 0$ either. We notice, however, that $S_{2,\mathrm{fl}}$ with the Lüscher–Weisz choice of coefficients $c_1=-1/12$ and $c_2=0$, has a rather simple structure,

$$\begin{aligned} \left. S_{2,\mathrm{fl}}\right| _\mathrm{LW}= & {} -2\int _0^\infty \mathrm{d}t \int \mathrm{d}^4x \sum _{\mu ,\nu }\mathrm{tr}\\&\times \left\{ L_\mu (t,x) \frac{1}{12} D_\mu ^2D_\nu ^{} G_{\nu \mu }(t,x)\right\} . \end{aligned}$$

To cancel this term is relatively straightforward. Starting from the lattice gradient force defined with the Lüscher–Weisz action, $\partial _{x,\mu }S_\mathrm{LW}$, we simply act with

50 $$\begin{aligned} 1+ \frac{1}{12} a^2 \nabla _\mu ^*\nabla _\mu ^{} \end{aligned}$$

on this gradient force, which yields the “Zeuthen flow” equation (5). The flow action $S_{2,\mathrm{fl}}$ for the Zeuthen flow does indeed vanish, i.e. we have successfully implemented $\mathcal O(a^2)$ improvement in the $4+1$-dimensional bulk.

### $\mathcal O(a^2)$ improvement of *E*(*t*, *x*)

We here consider only the simplest observable, namely the action density *E*(*t*, *x*) of Eq. (4) The two most popular lattice discretisations of *E*(*t*, *x*) are referred to as plaquette (pl) and clover (cl) definitions, respectively. They are either obtained from the Wilson plaquette action or based on the so called clover leaf definition of the field strength tensor,

51 $$\begin{aligned} G^{\text {cl}}_{\mu \nu }(t,x)= & {} \frac{1}{4a^2}\left( P_{\mu \nu }(t,x)+Q_{\mu \nu }(t,x) \right. \nonumber \\&\left. +R_{\mu \nu }(t,x)+S_{\mu \nu }(t,x) \right) _\mathrm{AH}, \end{aligned}$$

which uses the four plaquettes (32), (35)–(37) in the $\mu -\nu$ plane. The plaquette and clover lattice versions of *E*(*t*, *x*) are now given by

52 $$\begin{aligned} E^{\text {pl}}(t,x) \!=\! -\frac{a^{-4}}{2} \sum _{\mu ,\nu } \left[ \mathrm{tr}(P_{\mu \nu }(t,x) \!+\! P_{\mu \nu }(t,x)^\dagger ) \!-\!2N\right] ,\nonumber \\ \end{aligned}$$

and

53 $$\begin{aligned} E^{\text {cl}}(t,x) = -\frac{1}{2} \sum _{\mu ,\nu }\mathrm{tr}\{G^{\text {cl}}_{\mu \nu }(t,x) G^{\text {cl}}_{\mu \nu }(t,x)\}. \end{aligned}$$

Pushing the classical *a*-expansion of the plaquette $P_{\mu \nu }$ (32) to $\mathcal O(a^6)$ one obtains

$$\begin{aligned} E^{\text {pl}}(t,x)= & {} E^{\text {cont}}(t,x) + \frac{1}{24} a^2\sum _{\mu ,\nu } \left[ \mathrm{tr}(D_\mu G_{\mu \nu }(t,x))^2 \right. \nonumber \\&\left. +\,\mathrm{tr}(D_\nu G_{\mu \nu }(t,x))^2\right] \nonumber \\&-\frac{1}{4} a \sum _{\mu ,\nu }(\partial _\mu +\partial _\nu ) \mathrm{tr}(G_{\mu \nu }(t,x))^2 \nonumber \\&-\frac{1}{24} a^2 \sum _{\mu ,\nu } (2\partial _\mu ^2+2\partial _\nu ^2 + 3\partial _\mu \partial _\nu ) \mathrm{tr}(G_{\mu \nu }(t,x))^2 \nonumber \\&+\, \mathcal O(a^3), \end{aligned}$$

with the continuum limit $E^{\text {cont}}(t,x)$ given by Eq. (4). Proceeding in this way for all four plaquettes of the clover leaf we obtain the classical expansion

$$\begin{aligned} E^{\text {cl}}(t,x)= & {} E^{\text {cont}}(t,x) + \frac{1}{6} a^2\sum _{\mu ,\nu } \left[ \mathrm{tr}(D_\mu G_{\mu \nu }(t,x))^2 \right. \nonumber \\&\left. +\,\mathrm{tr}(D_\nu G_{\mu \nu }(t,x))^2\right] \nonumber \\&-\frac{1}{12} a^2 \sum _{\mu ,\nu }(\partial _\mu ^2+\partial _\nu ^2) \mathrm{tr}(G_{\mu \nu }(t,x))^2 + \mathcal O(a^4). \end{aligned}$$

Several remarks are in order. First, the *a*-expansion of the plaquette yields contributions at every order in *a*, whereas the symmetries of the clover definition imply only even powers of *a*. The odd powers of *a* could be eliminated by re-defining $E^\mathrm{pl}(t,x)$ as an average over the four plaquettes of the clover leaf, which, due to the trace operation, coincide with $\mathrm{tr}[P_{\mu \nu }(t,x)]$ for appropriately displaced arguments *x*. Second, note the total derivative terms which may appear at any order in *a*. Such terms do not contribute to the expectation value $\langle E(t,x)\rangle$, provided that the chosen set-up is translation invariant. This would e.g. be the case in a finite volume with periodic or twisted periodic boundary conditions, and thus in the limit of infinite volume. However, translation invariance no longer holds with either Dirichlet or Neumann conditions^5^ as required for the Schrödinger functional [13] or with open boundary conditions [14]. Similarly, when considering higher correlation functions such as the 2-point correlator of two fields *E*(*t*, *x*) total derivative terms cannot be ignored. We will here focus on the translation invariant case and from now on consider such total derivative terms negligible. This eliminates all the odd powers of *a* in the expansion of $E^\text {pl}(t,x)$. Hence, both discretisations are on equal footing and counterterms $\mathcal{O}_{2}$ for $E^{\text {pl}}$ and $E^{\text {cl}}$ are now easily identified as the $\mathcal O(a^2)$ coefficients in the classical expansion. Given both *a*-expansions we observe that the $\mathcal O(a^2)$ terms have the same structure, with the coefficients in the clover definition being larger by a factor of 4. In any case we observe that the linear combination

54 $$\begin{aligned} E^{\text {pl--cl}}(t,x) = \frac{4}{3} E^{\text {pl}}(t,x) -\frac{1}{3} E^{\text {cl}}(t,x), \end{aligned}$$

defines an $\mathcal O(a^2)$ improved observable for which $\mathcal{O}_2$ vanishes. An alternative $\mathcal O(a^2)$ improved definition of *E*(*t*, *x*) can be obtained from the action density of a tree-level improved lattice action such as the Lüscher–Weisz action (Eq. (6) with $c_0=5/3$, $c_1=-1/12$ and $c_{2,3}=0$). Here again, any ambiguity in the definition of a density from the action amounts to total derivative terms, which we consider negligible in the present context.

### Determination of $S_{2,b}$

In this subsection we list the gauge invariant local fields of dimension 6 which may appear in the boundary action $S_{2,b}$ of Symanzik’s effective action. Disregarding total derivative terms with respect to the space-time coordinates *x*, we find the following list of seven candidate counterterms:

55 $$\begin{aligned} O_1(x)= & {} \sum _{\mu ,\nu }\mathrm{tr}\{[D_\mu F_{\mu \nu }(x)] D_\mu F_{\mu \nu }(x)\}, \end{aligned}$$

56 $$\begin{aligned} O_2(x)= & {} \sum _{\mu ,\nu ,\rho }\mathrm{tr}\{[D_\mu F_{\nu \rho }(x)] D_\mu F_{\nu \rho }(x)\}, \end{aligned}$$

57 $$\begin{aligned} O_3(x)= & {} \sum _{\mu ,\nu ,\rho }\mathrm{tr}\{[D_\mu F_{\mu \nu }(x)] D_\rho F_{\rho \nu }(x)\}, \end{aligned}$$

58 $$\begin{aligned} O_4(x)= & {} \sum _{\mu ,\nu }\mathrm{tr}\{L_\mu (0,x) D_\nu F_{\nu \mu }(x)\}, \end{aligned}$$

59 $$\begin{aligned} O_5(x)= & {} \sum _{\mu }\mathrm{tr}\{L_\mu (0,x) L_\mu (0,x)\},\end{aligned}$$

60 $$\begin{aligned} O_6(x)= & {} \sum _{\mu ,\nu } \partial _t\mathrm{tr}\{G_{\mu \nu }(t,x)G_{\mu \nu }(t,x)\}\vert _{t=0},\end{aligned}$$

61 $$\begin{aligned} O_7(x)= & {} \sum _{\mu }\mathrm{tr}\{L_\mu (t,x)\partial _t B_\mu (t,x)\}\vert _{t=0}, \end{aligned}$$

where $F_{\mu \nu }$ denotes the field strength tensor of the fundamental gauge field.

Again we apply the field equations. The Yang–Mills flow equation implies

62 $$\begin{aligned} \partial _t G_{\mu \nu }(t,x) =\sum _\rho [D_\mu D_\rho G_{\rho \nu } - D_\nu D_\rho G_{\rho \mu }], \end{aligned}$$

so that, after taking into account the boundary condition $G_{\mu \nu }|_{t=0} = F_{\mu \nu }$, we have

63 $$\begin{aligned} O_6 + 4 O_3 = \text {total derivative}, \quad O_7 = O_4. \end{aligned}$$

This eliminates $O_{6,7}$. The field equation (46) is not useful here. However, a third field equation can be derived by varying the action at $t=0$ with respect to the fundamental gauge field $A_\mu (x)$. Technically this is best done by discretising only the flow time in the $4+1$-dimensional continuum action and taking the limit of continuous flow time in the end. The resulting field equation is^6^

64 $$\begin{aligned} \frac{1}{g^2} \sum _{\nu }D_\nu F_{\nu \mu }(x) = - L_\mu (0,x). \end{aligned}$$

This equation leads to the relations

65 $$\begin{aligned} O_5 = - \frac{1}{g^2} O_4, \quad O_3 = - g^2 O_4. \end{aligned}$$

At this point it is useful to recall the situation in the standard 4-dimensional theory [19]. In fact there is a 1-parameter family of $\mathcal O(a^2)$ improved actions, which, to tree-level, are parameterised by $x_p$ as follows:

66 $$\begin{aligned} \begin{array}{ll} c_0 = 5/3 -24 x_p,\quad c_1=-1/12 + x_p, \\ \quad c_2=x_p, \quad c_3=0. \end{array} \end{aligned}$$

Expanding the action classically, the free parameter $x_p$ is seen to multiply the counterterm $O_3$. The counterterm $O_3$ is thus redundant for the improvement of standard observables. In principle one may thus tune the coefficients (66) to achieve $\mathcal O(a^2)$ improvement of both standard and gradient flow observables. In practice, however, these coefficients define the gauge action used in the Monte-Carlo simulation and the corresponding effective coefficient of $O_3$ should be regarded as fixed. Therefore we choose $O_{1,2,4}$ as our basis of counterterms (cf. Sect. 3.6).

Finally, we remark that the use of the field equation (64) in the counterterm basis holds for counterterm insertions only up to contact terms, namely whenever the counterterm argument coincides with the location of some field in the correlation function under study. Such contact terms are thus absent for gradient flow observables localised at strictly positive flow times. However, we expect these relations to hold more generally, i.e. even if some fields in the correlation functions are defined at zero flow time. In this case we expect that the contact terms which make the difference are of the same form as the $\mathcal O(a^2)$ counterterms to the fields in the correlation function and therefore just redefine these counterterm coefficients. This parallels the discussion in Ref. [26] of on-shell $\mathcal O(a)$ improvement in lattice QCD with Wilson quarks.

### Summary of Section 3 and some practical considerations

Section 3 contains the main results of this paper and may appear rather technical. We therefore provide a short summary and comment on the practical implementation of the lattice counterterm $O_4$.

There is a natural way of interpreting the gradient flow as a $4+1$-dimensional local quantum field theory. The flow time *t* plays the role of the coordinate in the fifth dimension, which only takes on non-negative values ($t\ge 0$). The dynamics of the theory in the bulk ($t>0$) is completely fixed by the deterministic flow equation. The classical nature of the theory for $t>0$ allows one to implement the Symanzik improvement programme in a rather simple way: all $\mathcal O(a^2)$ cutoff effects produced by integrating the flow equation can be eliminated via a suitable discretisation of the flow equation, which can be determined by the classical expansion to $\mathcal O(a^2)$. Similar considerations allow one to define discretised flow observables that are free from $\mathcal O(a^2)$ lattice artefacts. The only remaining $\mathcal O(a^2)$ effects are generated by the action at the boundary $t=0$, and are genuine quantum effects. They correspond to the usual $\mathcal O(a^2)$ counterterms (55)–(57) in the 4-dimensional action affecting all lattice observables.

To implement an $\mathcal O(a^2)$ improved lattice action one first has to choose an $\mathcal O(a^2)$ improved 4-dimensional lattice gauge action which amounts to choosing coefficients $c_{0-3}$ in Eq. (6) appropriately. It is well known how $\mathcal O(a^2)$ improvement can be implemented at tree-level, and also to order $g_0^2$ in the case of the pure gauge theory [20]. In addition one needs to incorporate a lattice version of $O_4$ such as to cancel the insertion of $O_3$ on observables without changing the coefficients $c_{0-3}$.

To achieve this we remind the reader that the $4+1$-dimensional set-up is used only for the theoretical analysis, whereas in practice one integrates the gradient flow equation numerically and evaluates any observable such as *E*(*t*, *x*) along the flow. It turns out that the insertion of $O_4$ can be realised by a change in the initial condition at $t=0$ for the gradient flow equation. Since in this case $A_\mu (x)$ and $B_\mu (0,x)$ are not the same we need to fix the integration variables in the $4+1$-dimensional field theory. We choose to integrate over the fundamental gauge field $A_\mu (x)$ and the flow field $B_\mu (t,x)$ for $t>0$. Therefore on the lattice we choose to integrate over $U_\mu (x)$ and $V_\mu (t,x)$ for $t>0$. A shift in the initial condition can be implemented via

67 $$\begin{aligned} V_\mu (t,x)\vert _{t=0} = \mathrm{e}^{c_b g_0^2\partial _{x,\mu } S_\text {g}[U]} U_\mu (x), \end{aligned}$$

where $c_b$ is the free improvement coefficient, and $S_\text {g}[U]$ any 4-dimensional lattice action. In the $4+1$-dimensional formulation with $\varepsilon$-discretised flow time, the fields $V_\mu (0,x)$ and $U_\mu (x)$ only enter in the terms

$$\begin{aligned}&S_\text {g}[U] - 2 a^4 \sum _{x,\mu } \mathrm{tr}\{ L_\mu (0,x)[ a^{-1}(V_\mu (\varepsilon ,x)V_\mu (0,x)^\dagger - 1) \nonumber \\&\quad - \varepsilon X_\mu (0,x) ]\}, \end{aligned}$$

where $X_\mu (t,x)$ is, up to terms of $\mathcal O(\varepsilon )$, the RHS of the flow equation. Now we can trade all references to $V_\mu (0,x)$ into $U_\mu (x)$, that is our path integral variable. Using Eq. (67) we can write

68 $$\begin{aligned} \begin{array}{ll} V_\mu (\varepsilon ,x)V_\mu (0,x)^\dagger =&{} V_\mu (\varepsilon ,x)U_\mu (x)^\dagger \\ &{}-\,c_bg^2_0 \partial _{x,\mu }S_{\text{ g }}[U] + \cdots \end{array} \end{aligned}$$

where the dots represent higher order terms in the lattice spacing. Therefore the shift in the initial condition is equivalent (up to higher order corrections in *a*) to the insertion of the counterterm

$$\begin{aligned} 2c_ba^6\sum _{x} \hat{O}_4(x) = -2c_b a^3\sum _{x,\mu } \mathrm{tr}\{ L_\mu (0,x)(g_0^2\partial _{x,\mu } S_\text {g}[U] ) \}. \end{aligned}$$

This can be reinterpreted as the previous situation with standard boundary conditions, $V_\mu (0,x)=U_\mu (x)$, except for the extra $\hat{O}_4$ term in the action. Hence we have successfully traded the modified boundary conditions for the flow equation for the $O_4$ term in the lattice action. In the next section we will determine its coefficient $c_b$ at tree-level of perturbation theory.

## Perturbative analysis

In this section we will study the Symanzik $\mathcal O(a^2)$ improvement of the gradient flow in perturbation theory. This will allow us first to determine the improvement coefficient $c_b$ to tree-level. Second, the study of the Zeuthen flow both in small volumes and for different observables will allow us to check explicitly that the use of a tree-level improved action for the simulation together with the tree-level value^7^ of $c_b$, the Zeuthen flow and a classically improved definition of the observable yields expectation values that are free from $\mathcal O(a^2)$ effects at tree level. As observable we choose first *E*(*t*, *x*). The contributions by the action, flow and observable to the cutoff effects of $\langle E(t,x) \rangle$ at tree level have been computed recently [22, 27]. Here we will show that the $\mathcal O(a^2)$ tree-level cutoff effects are absent not only in infinite volume, but also in a finite volume with twisted periodic boundary conditions, where the additional scale *L* leads to more stringent tests. Second we will consider the connected correlation function for *E*(*t*, *x*)*E*(*s*, *y*) and show that $\mathcal O(a^2)$ improvement by the Zeuthen flow is also obtained in this case.

### Gauge fixing

In perturbation theory one parametrises the links in a neighbourhood of a classical configuration as follows:

69 $$\begin{aligned} \begin{array}{ll} U_\mu (x) &{}= \exp (ag_0A_\mu (x)),\\ V_\mu (t,x) &{}= \exp (ag_0B_\mu (t,x)). \end{array} \end{aligned}$$

Note that this standard convention implies a re-scaling of the fields,

70 $$\begin{aligned} A_\mu \longrightarrow g_0 A_\mu ,\quad B_\mu \longrightarrow g_0 B_\mu , \end{aligned}$$

compared to the preceding sections. In perturbation theory it is convenient to use gauge symmetry to simplify explicit computations. In the context of the gradient flow, gauge fixing is performed by studying the generalised flow equation

$$\begin{aligned} \begin{array}{ll} \partial _t B_\mu ^{(\alpha )}(t,x) &{}= D_\nu ^{(\alpha )} G_{\nu \mu }^{(\alpha )}(t,x) + \alpha D_\mu ^{(\alpha )}\partial _\nu B_\nu ^{(\alpha )}(t,x),\\ \quad B_\mu ^{(\alpha )}(0,x) &{}= A_\mu (x). \end{array} \end{aligned}$$

The superscript ${(\alpha )}$ serves as a reminder that covariant derivatives and field strength are made of the modified flow field $B_\mu ^{(\alpha )}(t,x)$, i.e. the solution of the above equation. Note that the original flow equation is recovered by setting $\alpha =0$. The key observation is that gauge invariant observables are independent of $\alpha$ [1, 3, 24]. In order to see this, one only has to check that the gauge transformation

71 $$\begin{aligned} B_\mu = \Lambda B_\mu ^{(\alpha )}\Lambda ^{-1} +1/g_{0}^{2} \Lambda \partial _\mu \Lambda ^{-1} , \end{aligned}$$

where

72 $$\begin{aligned} \partial _t\Lambda = \alpha g_0 \Lambda \partial _\mu B_\mu ;\quad \Lambda \big |_{t=0} = 1, \end{aligned}$$

transforms a solution of the flow equation with arbitrary $\alpha$ into one with $\alpha =0$.

On the lattice the procedure is completely analogous. We consider the generalised flow equation

73 $$\begin{aligned} \begin{array}{ll} &{}a^2\partial _t V_\mu ^\Lambda (t,x) = \{-g_{0}^{2} \partial _{x,\mu } S_\text {g}(V^\Lambda ) \\ &{}\quad +\, a^3\nabla _\mu ^{\Lambda }[\Lambda (t,x)^\dagger \partial _t \Lambda (t,x)] \} V_\mu ^\Lambda (t,x), \end{array} \end{aligned}$$

or, for the case of the Zeuthen flow,

$$\begin{aligned} \begin{array}{ll} &{} a^2\partial _t{V}_\mu ^\Lambda (t,x) = \Bigg \{ -g_0^2 \left( 1+\frac{a^2}{12} \nabla _\mu ^{\Lambda *} \nabla _\mu ^\Lambda \right) \partial _{x,\mu } S_\text {LW}(V^\Lambda ) \\ &{}\quad +\, a^3\nabla _\mu ^\Lambda [\Lambda (t,x)^\dagger \partial _t \Lambda (t,x)] \Bigg \}V_\mu ^\Lambda (t,x), \end{array} \end{aligned}$$

with initial condition $V_\mu ^\Lambda (0,x) = U_\mu (x)$. One then easily verifies that the gauge transformation

74 $$\begin{aligned} V_\mu (t,x) = \Lambda (t,x)V_\mu ^\Lambda (t,x)\Lambda (t,x+a\hat{\mu })^\dagger , \end{aligned}$$

transforms a solution with an arbitrary function $\Lambda (t,x)$ into one with $\Lambda =1$. A natural choice for the function $\Lambda (t,x)$ then is given as the solution of the equation,

75 $$\begin{aligned} \Lambda (t,x)^\dagger \partial _t \Lambda (t,x) = \alpha g_0 \partial ^*_\mu B_\mu (t,x),\quad \Lambda \big |_{t=0} = 1. \end{aligned}$$

Note that this is a particular application of the $4+1$-dimensional gauge transformations described in Sect. 2.3 and it is thus clear that gauge invariant observables remain unaffected by the choice of $\alpha$. This can be turned around to provide checks on the correctness of a given calculation. In the following we drop the indices $(\alpha )$ (or $\Lambda$) from the fields and we will quote any intermediate results in Feynman gauge ($\alpha =1$). Some elements used for our checks of gauge parameter independence are given in Appendix C.

### Determination of $c_b$ to tree level

We first assume that the lattice is infinitely extended and expand the general class of actions, Eq. (6), to leading order in the coupling,^8^

76 $$\begin{aligned} S_\text {g}[U;\{c_i^{(a)}\}]= & {} \frac{1}{2} \sum _{\mu ,\nu }\int _p \tilde{A}_\mu ^a(-p) K_{\mu \nu }^{(a)}(p;\lambda ) \tilde{A}_\nu ^a(p) \nonumber \\&+\, \mathcal O(g_0), \end{aligned}$$

where $\lambda$ is a gauge fixing parameter and explicit expressions for the lattice kernels, $K_{\mu \nu }^{(a)}(p;\lambda )$, are given in Appendix A.

Similarly, the flow equation contains the gradient of a lattice action which, to leading order in the coupling, is parameterised by another action kernel, $K^{(f)}_{\mu \nu }(p;\alpha )$. The flow equation to this order then takes the form of the heat equation,

77 $$\begin{aligned} \partial _t {\tilde{B}_\mu ^a(t,p)} = -\sum _\nu K_{\mu \nu }^{(f)}(p;\alpha ) \tilde{B}_\nu ^a(t,p). \end{aligned}$$

The initial condition for the flow equation Eq. (67) reads to leading order in the fields,^9^

$$\begin{aligned} \tilde{B}_\mu (0,p) = \sum _\nu [\delta _{\mu \nu } + a^2c_b K_{\mu \nu }^{(i)}(p;0) ] \tilde{A}_\nu (p), \end{aligned}$$

where $K_{\mu \nu }^{(i)}$ is yet another action kernel. No gauge fixing term is required here, so that the gauge parameter is set to zero. The linearised flow equation (77) can now be solved easily

78 $$\begin{aligned} \tilde{B}_\mu ^a(t,p) = \sum _{\nu ,\rho } H_{\mu \nu }(t,p;\alpha ) [ \delta _{\nu \rho } + a^2c_b K_{\nu \rho }^{(i)}(p;0) ] \tilde{A}_\rho ^a(p), \end{aligned}$$

where $H_{\mu \nu }$ is the heat kernel given by

79 $$\begin{aligned} H_{\mu \nu }(t,p;\alpha ) = \exp (-t K^{(f)}(p;\alpha ))_{\mu \nu }. \end{aligned}$$

Note that we have used here $K^{(f)}(p;\alpha )$ as a matrix with respect to the Lorentz indices and the exponential has to be taken of that matrix. In the following we will often make use of such a matrix notation, in order to avoid an abundance of Lorentz indices.

Finally, the observable *E*(*t*, *x*), being an action density, can be parameterised by a further lattice action kernel, $K^{(o)}(p,0)$, with gauge fixing parameter set to zero. To this order we then obtain for the expectation value

$$\begin{aligned} \langle E(t,x) \rangle= & {} \frac{N^2-1}{2} g_0^2 \int _p \mathrm{Tr}\{ K^{(o)}(p;0)\,\bar{D}(p,t,t;\alpha ,\lambda )\,\} \\&+\, \mathcal O(g_0^4), \end{aligned}$$

where the trace is over Lorentz indices only and the gauge field propagator at positive flow time is defined by

$$\begin{aligned} \langle \tilde{B}_{\mu }^a(s,p)\tilde{B}_\nu ^b(t,q) \rangle = (2\pi )^4 \delta ^{(4)} (p+q)\delta ^{ab}\bar{D}_{\mu \nu }(p,s,t;\alpha ,\lambda ) . \end{aligned}$$

Due to the relation (78), this propagator depends implicitly on both gauge parameters, $\alpha$ and $\lambda$, of the flow equation and of the action, respectively. Introducing the standard 4-dimensional gauge field propagator

80 $$\begin{aligned} \langle \tilde{A}_{\mu }^a(p)\tilde{A}_\nu ^b(q) \rangle = (2\pi )^4 \delta ^{(4)} (p+q)\delta ^{ab} D_{\mu \nu }(p;\lambda ), \end{aligned}$$

this propagator is the matrix inverse of the action kernel,81and the gauge fixing parameter $\lambda$ must be non-zero for the inverse to exist. Using these ingredients, the gauge field propagator at positive flow time can now be written as follows:82where we have denoted the matrix transpose by the superscript T.

In summary, the choices of action, flow and observable discretisation correspond to the choice of three action kernels. Finally the shift in the initial condition is encoded in a fourth choice of kernel. Explicit expressions for some popular choices of kernels are given in Appendix C.

In order to obtain the leading order cutoff effects we now expand the kernels as follows:

83 $$\begin{aligned} K(p;\lambda ) = K^\mathrm{cont}(p;\lambda ) + a^2R(p;\lambda ) + \mathcal O(a^4), \end{aligned}$$

where the continuum kernel is given by

84 $$\begin{aligned} K_{\mu \nu }^\mathrm{cont}(p;\lambda ) = p^2\delta _{\mu \nu } - (1-\lambda )p_\mu p_\nu . \end{aligned}$$

Using the continuum kernel only and neglecting cutoff effects we thus obtain the well-known continuum result in infinite volume,

85 $$\begin{aligned} \begin{array}{ll} \langle E(t,x) \rangle =&{} g_0^2\mathcal E_0^\mathrm{cont}(t) + \mathcal O(g_0^4,a^2), \\ \mathcal E_0^\mathrm{cont}(t) =&{} \frac{3(N^2-1)}{128 \pi ^2 t^2} . \end{array} \end{aligned}$$

Explicit expressions for the correction terms $R_{\mu \nu }(p;\lambda )$ are given in Appendix C. In order to compute the leading correction to the propagator $D_{\mu \nu }(p;\lambda )$ and to the heat kernel $H_{\mu \nu }(t,p;\alpha )$ it is convenient to work in Feynman gauge ($\lambda =\alpha =1$), since in this case $K_{\mu \nu }^\mathrm{cont}(p;1)$ is proportional to $\delta _{\mu \nu }$. Working in a general gauge is, however, not much more difficult and serves as a check that the gauge dependence actually cancels in the final evaluation of the observable. A few technical details pertaining to such a check are given in Appendix C.

In the following we will use Feynman gauge and remove the gauge parameters as arguments of the action and flow kernels. We will also omit them in the kernels for the observable and initial conditions however, with the understanding that they must be set to zero in these cases. In Feynman gauge ($\lambda =\alpha =1$) it is straightforward to check that

86a $$\begin{aligned}&D_{\mu \nu }(p) = \frac{1}{p^2}\left[ \delta _{\mu \nu } - \frac{a^2}{p^2}R_{\mu \nu }(p) \right] + \mathcal O(a^4)\end{aligned}$$

86b $$\begin{aligned}&H_{\mu \nu }(t,p) = \mathrm{e}^{-tp^2}\left[ \delta _{\mu \nu } - a^2tR_{\mu \nu }(p) \right] + \mathcal O(a^4), \end{aligned}$$

and finally, putting all the pieces together and after some algebra, we get

87 $$\begin{aligned} \begin{array}{ll} \mathcal E_0(t) &{}= \mathcal E_0^\mathrm{cont}(t)\left\{ 1 + \frac{a^2}{t}\left[ (d_1^{(o)}-d^{(a)}_1)J_{4,-2}\right. \right. \\ &{}\quad \left. \left. +\, (d_2^{(o)}-d^{(a)}_2+2c_b)J_{2,0}\right. \right. \\ &{}\quad \left. \left. -\, 2 d_1^{(f)}J_{4,0} -2 d_2^{(f)}J_{2,2}\right] \right\} \\ &{}\quad +\, \mathcal O(a^4), \end{array}\nonumber \\ \end{aligned}$$

where the constants $J_{n,m}$ are defined by

88 $$\begin{aligned} J_{n,m} = t^{(n+m)/2}\frac{\int _{-\infty }^\infty d^4p \mathrm{e}^{-2tp^2}\, (p^n)(p^m)}{\int _{-\infty }^\infty d^4p \mathrm{e}^{-2tp^2}}, \end{aligned}$$

and

89 $$\begin{aligned} p^n = \left\{ \begin{array}{ll} \sum _\mu (p_\mu )^n &{}\quad n>0 \\ \left[ \sum _\mu (p_\mu )^{|n|}\right] ^{-1} &{}\quad n<0 \\ \end{array} \right. \,. \end{aligned}$$

In fact it is straightforward to evaluate the integrals, with the result

90 $$\begin{aligned} \begin{array}{ll} J_{4,-2} = 1/2,\quad J_{2,0} = 1,\\ J_{4,0} = 3/4,\quad J_{2,2} = 3/2. \end{array} \end{aligned}$$

The coefficients $d_{1,2}^{(a,o,f)}$ must be independent of the gauge parameters $\alpha$ and $\lambda$ and we have checked this explicitly. Their values depend on the choices made for the various kernels. For example, for a general action of the form Eq. (6) we have

91a $$\begin{aligned} d_1= & {} -\frac{1}{12}-\frac{2}{3}c_1+\frac{2}{3}c_2+\frac{2}{3}c_3,\end{aligned}$$

91b $$\begin{aligned} d_2= & {} -\frac{1}{3}c_1 -\frac{2}{3}c_2-\frac{2}{3}c_3. \end{aligned}$$

Table 1 summarises the values of the coefficients $d_{1,2}^{(a,o,f)}$ for the most common choices. It is easy to see that the use of the Zeuthen flow together with the tree-level improved Lüscher–Weisz action and any classically improved discretisation for the observable (see Sect. 3.4) has no tree-level $\mathcal O(a^2)$ cutoff effects as long as $c_b=0$. Therefore, to tree-level, the Lüscher–Weisz action ($c_1=-1/12, c_2=0$) produces tree-level improved results for gradient flow observables. For the case of a generalised tree-level improved action Eq. (66) we have to choose

92 $$\begin{aligned} c_b = -\frac{1}{2}x_p, \end{aligned}$$

in order to obtain tree-level improvement.

**Table 1:** Values of the coefficients in the $\mathcal O(a^2)$ terms of $t^2\langle E(t,x) \rangle$ in infinite volume. The one-parameter family of tree-level improved actions corresponds to the choice of coefficients Eq. (66), the Lüscher–Weisz tree-level improved action being the particular choice with $x_p=0$

Discretisation	$d_1$	$d_2$
Plaquette	$-1/12$	0
Lüscher–Weisz	$-1/36$	1 / 36
$\frac{4}{3}$ Plaquette $-\frac{1}{3}$ Clover	$-1/36$	1 / 36
One-parameter tree-level improved	$-1/36$	$1/36-x_p$
Clover	$-1/4$	$-1/12$
Zeuthen	0	0

As the reader can see, besides the Zeuthen flow there seem to be many ways to cancel the tree-level $\mathcal O(a^2)$ effects (see also [22]), as these are encoded in a single term, once the numerical values of (90) and for $d_{1,2}$ (cf. Table 1) are inserted into Eq. (87). We are thus led to look for more stringent tests of $\mathcal O(a^2)$ improvement by looking at a variety of observables and/or kinematics. After all, rather than improving a particular observable in a specific situation (e.g. in infinite volume), Symanzik improvement is designed to work for any observable in both finite and infinite volume.

### Twisted periodic boundary conditions

A stringent test of our computations can be made when studying $t^2\langle E(t,x) \rangle$ in a finite volume. Due to the presence of a new scale *L*, the cutoff effects will in general depend on the dimensionless ratio $c=\sqrt{8t}/L$. Improvement requires that the tree-level cutoff effects vanish *for all values of*$c=\sqrt{8t}/L$.

As a finite volume renormalisation scheme, we will use twisted boundary conditions for our gauge field. In this set-up, the gauge field changes by a gauge transformation when displaced by a period. Gauge invariant quantities are still periodic, and the absence of zero-modes turns out to be very convenient for perturbative computations. The gradient flow has already been studied in this set-up, and we will not give many details here but refer the interested reader to Ref. [15] and the references cited therein.

We will only need the perturbative expression of $\langle E(t,x)\rangle$ to leading order, given by

93a $$\begin{aligned} \langle E(t,x)\rangle = g_0^2\mathcal E_0(t,c) + \mathcal O(g_0^4) \end{aligned}$$

with93b

Note that the expression is almost identical to the infinite volume one, except that the momentum integral has been substituted by a sum (hardly a surprise). The particularities of the twisted boundary conditions are hidden in the sum and momentum symbols. First notice that the momentum (with capital letters $P_\mu$) can be uniquely decomposed as

94 $$\begin{aligned} P_\mu = \frac{2\pi n_\mu }{L} + \frac{2\pi \tilde{n}_\mu }{NL}, \end{aligned}$$

with $$n_\mu = 0,\dots ,L/a-1$$ and

95 $$\begin{aligned} \tilde{n}_\mu = \left\{ \begin{array}{ll} 0, &{}\quad \text { if } \mu = 0,3, \\ 0,\dots ,N-1, &{}\quad \text { if } \mu = 1,2, \end{array}\right. \end{aligned}$$

i.e. there is the usual space-momentum, but in the directions of the twisted plane $x_1-x_2$ the momentum $P_\mu$ lives in an apparently larger lattice of physical extent *NL*. Finally the sum symbol $\sum ^{\prime }_P$ means sum both over $n_\mu$ and $\tilde{n}_\mu$, but without the terms with $\tilde{n}_1 = \tilde{n}_2 = 0$. In particular the sum has no term with a zero total momentum. Notice that the colour factor $N^2-1$ is produced by the sum over $\tilde{n}_\mu$.

The algebra is very similar to the one of the previous section, with the important difference that now the sums actually depend on the dimensionless ratio $c=\sqrt{8t}/L$. In fact fixing the flow time in units of the volume in this way we get

96 $$\begin{aligned} \begin{array}{ll} \mathcal E_0(t,c) =&{} \mathcal E_0^\mathrm{cont}(t,c) \\ &{}\times \bigg \{ 1 + \frac{a^2}{t^2}[ (d_1^{(o)}-d^{(a)}_1)\mathcal J_{4,-2}(c) \\ &{} + (d_2^{(o)}-d^{(a)}_2+2c_b)\mathcal J_{2,0}(c) \\ &{} - 2 d_1^{(f)}\mathcal J_{4,0}(c) -2 d_2^{(f)}\mathcal J_{2,2}(c) ]\bigg \}\\ &{}+\mathcal O(a^4), \end{array}\nonumber \\ \end{aligned}$$

where

97 $$\begin{aligned} \begin{array}{ll} \mathcal E_0^\mathrm{cont}(t,c) &{}= \frac{3c^4}{128t^2} \vartheta _3^2(0|\imath \pi c^2) \\ &{}\quad \times [ \vartheta _3^2(0|\imath \pi c^2/N^2) - \vartheta _3^2(0|\imath \pi c^2) ], \end{array} \end{aligned}$$

and the third Jacobi theta function reads

98 $$\begin{aligned} \vartheta _3\left( z|\tau \right) = \sum _n \mathrm{e}^{\imath \pi \tau n^2} \mathrm{e}^{2\imath n z}. \end{aligned}$$

Finally the functions $\mathcal J_{i,j}(c)$ are given by^10^

99 $$\begin{aligned} \begin{array}{ll} &{}\mathcal J_{i,j}(c) = \left( \frac{c\pi }{\sqrt{2}}\right) ^{i+j} \\ &{}\quad \times \dfrac{\sum ^{\prime }_n \mathrm{e}^{-c^2\pi ^2(n + \tilde{n}/N)^2}\, (n+\tilde{n}/N)^i (n+\tilde{n}/N)^j }{\vartheta _3^2(0|\imath \pi c^2)[ \vartheta _3^2(0|\imath \pi c^2/N^2) - \vartheta _3^2(0|\imath \pi c^2) ]}. \end{array} \end{aligned}$$

**Fig. 2:**
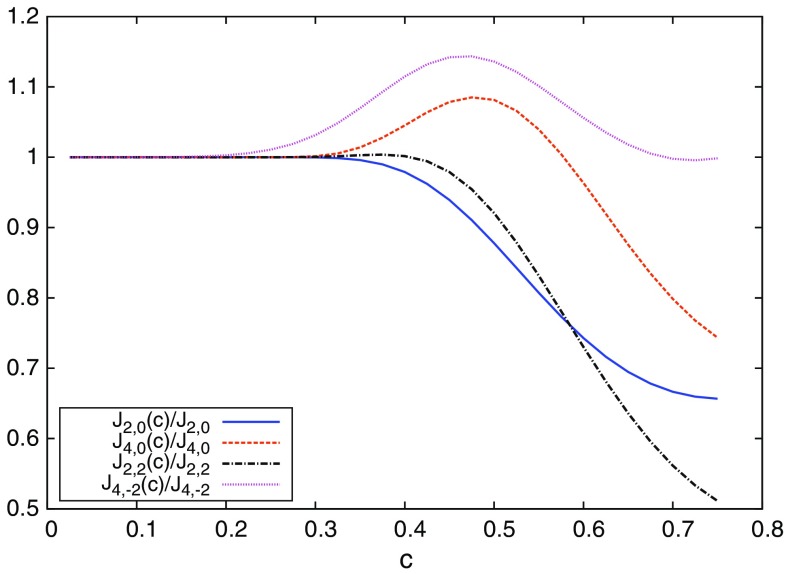
Ratio of the finite volume functions $\mathcal J_{i,j}(c)$ (Eq. 90) over the infinite volume predictions $J_{i,j}$ (Eq. 99). When $c>0.2$ there are significant differences between them. Moreover, the different functions $\mathcal J_{i,j}(c)$ are in general linearly independent

In the limit $c=\sqrt{8t}/L\rightarrow 0$, we recover the expressions of the infinite volume, in particular

100 $$\begin{aligned} \lim _{c\rightarrow 0} \mathcal J_{i,j}(c) = J_{i,j}, \end{aligned}$$

but for non-zero *c* the functions $\mathcal J_{i,j}(c)$ are in general linearly independent (see Fig. 2). The coefficients $d_{1,2}^{(a,o,f)}$ are still the same, and the reader can check that the tree-level $\mathcal O(a^2)$ cutoff effects given by expression Eq. (96) vanish for all values of *c* when one uses our improved set-up (i.e. Lüscher–Weisz action, Zeuthen flow and Lüscher–Weisz observable). Any other choice of improved action together with the appropriate choice of $c_b$ also does the work. For this to happen it is crucial that the flow coefficients $d_{1,2}^{(f)}$ are both zero, since the functions $\mathcal J_{4,0}(c)$ and $\mathcal J_{2,2}(c)$ are linearly independent. In particular it is easy now to check that the so called Symanzik flow in the literature [28] or any set of coefficients in [22] does *not* remove the tree-level cutoff effects in finite volume. For the Zeuthen flow both coefficients do identically vanish, so that $\mathcal O(a^2)$ effects are indeed removed as expected on theoretical grounds.

### The connected 2-point function of *E*(*t*, *x*)

Further tests of the Zeuthen flow can be obtained by considering different operators at positive flow time. In particular, we now consider the 2-point function of *E*(*t*, *x*) in a periodic box of size *L* with twisted periodic boundary conditions (i.e. the same set-up as above),

101 $$\begin{aligned} \langle E(t,x)E(s,0)\rangle _c = \langle E(t,x)E(s,0)\rangle - \langle E(t,x)\rangle \langle E(s,0)\rangle . \end{aligned}$$

Introducing the two dimensionless parameters

102 $$\begin{aligned} c = \frac{\sqrt{8t}}{L};\quad d = \frac{\sqrt{8s}}{L}, \end{aligned}$$

we write the leading order result in the form

103 $$\begin{aligned} \langle E(t,x)E(s,0)\rangle _c = g_0^4\mathcal M(t,s,c,d;x) + \mathcal O(g_0^6), \end{aligned}$$

with

104 $$\begin{aligned} \begin{array}{ll} &{}\mathcal M(t,s,c,d;x) = \frac{c^4 d^4}{1024t^2 s^2} \\ &{}\quad \times \sum _{P, Q} \mathrm{e}^{\imath (P+Q)x} \,\mathrm{Tr}\{ K(P,Q) H(t,P) \\ &{}\quad \times D(P) H(s,-P)^\mathrm{T}K(Q,P) \\ &{}\quad \times H(t,-Q)^\mathrm{T} D(Q)^\mathrm{T} H(s,Q) \}. \end{array}\nonumber \\ \end{aligned}$$

The generalised kernel *K*(*P*, *Q*) encodes the discretisation of the observable. Up to terms of $\mathcal O(a^2)$ it is given by

105 $$\begin{aligned} K_{\mu \nu }(P,Q) = K_{\mu \nu }^\mathrm{cont}(P,Q)+ \mathcal O(a^2), \end{aligned}$$

with the continuum kernel given by

106 $$\begin{aligned} K_{\mu \nu }^\mathrm{cont}(P,Q)= & {} \sum _{\rho }P_\rho Q_\rho \delta _{\mu \nu } - P_\mu Q_\nu . \end{aligned}$$

The finite volume calculation for $\langle E(t,x)\rangle$ has taught us that the $\mathcal O(a^2)$ contributions of the flow have to cancel by themselves, i.e. a cancellation with other $\mathcal O(a^2)$ contributions by the action or the observable is not possible, due to the linear independence of the momentum sums. In order to assess the improvement of the Zeuthen flow it is therefore enough to focus on these $\mathcal O(a^2)$ contributions. Using again the Feynman gauge for flow and action, we obtain $\mathcal O(a^2)$ terms from the flow of the form

$$\begin{aligned}&-a^2\frac{c^4 d^4}{1024t^2s^2} \sum _{P, Q} \mathrm{e}^{\imath (P+Q)x} \mathrm{e}^{-(t+s)\left( P^2+Q^2\right) } \frac{1}{P^2 Q^2} \\&\quad \times \,\mathrm{Tr}\{ K^\mathrm{cont}(P,Q) (tR(P)+sR(P)^\mathrm{T})\\&\quad \times K^\mathrm{cont}(Q,P)\}, \end{aligned}$$

and a second term with a similar structure. In both cases it is useful to note the property of the kernel that

107 $$\begin{aligned} K^\mathrm{cont}(P,Q) = T(Q) K^\mathrm{cont}(P,Q) T(P), \end{aligned}$$

where *T*(*P*) is the transverse projector,

108 $$\begin{aligned} T_{\mu \nu }(P) = \delta _{\mu \nu } - \frac{P_\mu P_\nu }{P^2}. \end{aligned}$$

The $\mathcal O(a^2)$ correction to the Zeuthen flow kernel, $R^Z(P)$, has the nice property that

109 $$\begin{aligned} T(P) R^Z(P) T(P) = 0. \end{aligned}$$

Hence we can conclude that the Zeuthen flow does not contribute any $\mathcal O(a^2)$ effects to this 2-point function either. Due to the different Lorentz index structure of this case compared to the simpler case of *E*(*t*, *x*), and to the fact that now, in general, the cutoff effects are functions of two variables (*c*, *d*), this test imposes further constraints on the possible improvement solutions. In particular, the so called *chair flow* in [27], which happens to also cancel the $\mathcal O(a^2)$ effects of $\langle E(t,x)\rangle$*in a finite volume*, can be shown to produce $\mathcal O(a^2)$ contributions to the 2-point function considered here.

## Conclusions and outlook

We have systematically investigated the structure of $\mathcal O(a^2)$ effects in flow quantities using Symanzik’s approach applied to the $4+1$-dimensional local formulation of the theory. Improvement to $\mathcal O(a^2)$ for gradient flow quantities appears to be easier than one might have thought, mainly due to the classical nature of the gradient flow equation. In particular the classical *a*-expansion is sufficient to obtain the counterterms for both local composite operators at positive flow time and the action in the $4+1$-dimensional bulk (i.e. due to the absence of loops in the bulk, no new counterterms are generated).

Our main results are summarised in the Zeuthen flow equation (5) and the improved lattice definitions of the observable *E*(*t*, *x*), either as linear combination of clover and plaquette definitions (54) or as the action density of the tree-level improved Lüscher–Weisz action. We have shown that the integration of this Zeuthen flow equation and the evaluation of classically improved observables do not produce any $\mathcal O(a^2)$ effects to any order in the coupling or, indeed, non-perturbatively. At this point it is important to remark that although the analysis has been performed in the context of pure gauge theories, due to the classical nature of the flow equation, the aforementioned results are still valid in QCD or if any number of fermions in any representations are coupled to our gauge field. In the particular case of the pure gauge theory the only $\mathcal O(a^2)$ effects originate either from the 4-dimensional lattice action or from the additional counterterm parameterised by $c_b$ in the modified initial condition (67). Tree-level $\mathcal O(a^2)$ improvement is achieved with the Lüscher–Weisz gauge action and $c_b=0$.

We have explicitly checked that the proposed Zeuthen flow equation does not generate any $\mathcal O(a^2)$ contribution to tree level for a variety of gluonic observables (different observables in arbitrary volumes). In doing so, we have shown that other proposals of the literature to improve the gradient flow (i.e. the $\tau$-shift in [21], the coefficients in [22] or the chair flow in [27]) in fact do produce $\mathcal O(a^2)$ effects in some of the considered observables. In this sense, these proposals only produce vanishing $\mathcal O(a^2)$ cutoff effects in some particular situations (i.e. $\langle E(t,x)\rangle$ in infinite volume), and this cancellation should be regarded as accidental, and not as improvement.

Our results can be extended in various directions. First, it appears straightforward to extend the classical *a*-expansion to further observables, for example the energy-momentum tensor. When considering *n*-point correlation functions of such observables with $n>1$ or if boundary conditions do not respect translation invariance in some directions (as is the case with SF and open boundary conditions), some additional work is required to also eliminate total derivative terms which may contribute at any order in *a*. We also note that the improvement of observables and the flow equation are conceptually separate from the $\mathcal O(a^2)$ effects at $t=0$. It is therefore conceivable to push the expansion further, in order to also cancel terms at $\mathcal O(a^4)$. It is not clear how complicated this would be for the flow equation, but it is certainly an option for observables. However, one should be aware that higher order improvement would typically render these observables less local in lattice units. Another natural generalisation would be the extension of our work to include fermions and the fermionic flow equation, introduced in Ref. [4].

## Conventions and notation

We will use the summation convention for colour indices

110 $$\begin{aligned} a,b,\ldots = 1,\dots , N^2-1 , \end{aligned}$$

but not for space-time indices $\mu ,\nu , \ldots$, as this may lead to confusion in the discussion of lattice artefacts. Trace over colour indices will be denoted by $\mathrm{tr}$ (lower case), while trace over Lorentz indices will be denoted with the symbol $\mathrm{Tr}$ (upper case).

*SU*(*N*) gauge fields live in the Lie algebra $\mathfrak {su}(N)$ and are traceless antihermitian $N\times N$ matrices. Any element $X\in \mathfrak {su}(N)$ of this algebra can be written as $X = X^a T^a$ where the components $X^a$ are real numbers and the generators $T^a$ are themselves antihermitian $N\times N$ matrices chosen to obey the normalisation

111 $$\begin{aligned} \mathrm{tr}(T^aT^b) = -\frac{1}{2}\delta _{ab}. \end{aligned}$$

On the lattice the links $U_\mu (x)$ belong to the gauge group *SU*(*N*). For an arbitrary function of the link variables $f(U_\mu (x))$, the Lie-algebra valued derivative is given by

$$\begin{aligned} \partial _{x,\mu } f(U_\mu (x)) = T^a \partial _{x,\mu }^a f(U_\mu (x)) = T^a\frac{\mathrm{d}}{\mathrm{d}\epsilon }f(\mathrm{e}^{\epsilon T^a}U_\mu (x)) \bigg |_{\epsilon =0}. \end{aligned}$$

Fourier transformations on an infinite lattice with lattice spacing *a* are defined as

112 $$\begin{aligned} A_\mu (x)= & {} \int _p \mathrm{e}^{\imath px+\imath p_\mu a/2}\tilde{A}_\mu (p), \end{aligned}$$

where

113 $$\begin{aligned} \int _p = \int _{-\pi /a}^{\pi /a} \frac{\mathrm{d}^4p}{(2\pi )^4}. \end{aligned}$$

On a hypercubic lattice of volume $L^4$ we define

114 $$\begin{aligned} A_\mu (x) = \frac{1}{L^4} \sum _n \mathrm{e}^{\imath px+\imath p_\mu a/2}\tilde{A}_\mu (p), \end{aligned}$$

with $p_\mu =2\pi n_\mu /L$ and $n_\mu = 0,\dots ,L/a-1$. It is convenient to introduce the lattice derivatives

115 $$\begin{aligned} \partial _\mu \phi (x)= & {} \frac{\phi (x+a\hat{\mu }) - \phi (x)}{a}, \end{aligned}$$

116 $$\begin{aligned} \partial _\mu ^* \phi (x)= & {} \frac{\phi (x) - \phi (x-a\hat{\mu })}{a}, \end{aligned}$$

and also the covariant derivatives given by

117 $$\begin{aligned} a\nabla _\mu f(x)= & {} U_\mu (x)f(x+a\hat{\mu })U_\mu (x)^\dagger - f(x), \end{aligned}$$

118 $$\begin{aligned} a\nabla _\mu ^* f(x)= & {} f(x) - U_\mu (x-a\hat{\mu })^\dagger f(x-a\hat{\mu })U_\mu (x-a\hat{\mu }).\nonumber \\ \end{aligned}$$

Along the work we use the following definitions of the lattice momenta:

119 $$\begin{aligned} \hat{p}_\mu= & {} \frac{2}{a}\sin (ap_\mu /2), \end{aligned}$$

120 $$\begin{aligned} \mathring{p}_\mu= & {} \frac{1}{a}\sin (ap_\mu ), \end{aligned}$$

121 $$\begin{aligned} \hat{c}_\mu= & {} \cos (ap_\mu /2). \end{aligned}$$

## Absence of odd powers of *a* in the classical expansion of $S_\text {fl}[V]$

In this appendix we demonstrate that the apparent presence of odd powers of *a* in the classical expansion (44) is an artefact of the way the expansion was set up. In particular, we will show that Symanzik’s effective action for the flow action only contains terms which are even powers of *a*.

### Re-expanding around the midpoint of the link

Indeed, the expansion about *x* does not account for the fact that the equation is derived for a given link variable $V_\mu (t,x)$, relating the lattice points *x* and $x+a\hat{\mu }$. Odd powers of *a* in the expansion are due to this asymmetric treatment, as can be shown explicity to all orders in *a* for the LHS of the flow equation, Eq. (40). First, we define the unitary matrices $\Omega _\mu (t,x)$ as the parallel transporters along the half link from *x* to the midpoint $\tilde{x} = x+ \frac{1}{2} a\hat{\mu }$,

122 $$\begin{aligned} \Omega _\mu (t,x) = \mathcal{P} \exp \left\{ a \int _{\frac{1}{2}}^{1} \mathrm{d}u\, B_\mu \left( t,z(u)\right) \right\} , \end{aligned}$$

i.e. compared to the path-ordered exponential $V_\mu (t,x)$ (30) we here only integrate over second half of the path parameterising the link. Now we can perform the parallel transport to the midpoint $\tilde{x}$, defining

123 $$\begin{aligned} F_\mu (t,x)= & {} \Omega _\mu (t,x) \tilde{F}_\mu (t,\tilde{x}) \Omega _\mu (t,x)^{-1}, \end{aligned}$$

and, analogously, $\tilde{L}(t,\tilde{x})$, such that the term in the lattice flow action density,

124 $$\begin{aligned} \mathcal{L}^{(\mu )} = \mathrm{tr}\{ L_\mu (t,x) F_\mu (t,x) \} = \mathrm{tr}\{ \tilde{L}_\mu (t,\tilde{x})\tilde{F}_\mu (t,\tilde{x}) \}, \end{aligned}$$

can be expressed in terms of fields defined at the midpoint. To obtain the expansion in powers of *a* about $\tilde{x}$ one may simply re-expand the expansion about *x* obtained previously. The parallel transporters $\Omega _\mu (t,x)$ then merely render the Taylor expansion covariant. Proceeding in this way yields, for the first term of $\tilde{F}_\mu (t,\tilde{x})$,

125 $$\begin{aligned}&a^{-1}\Omega _\mu (t,x)^\dagger [\partial _t V_\mu (t,x)]V_\mu (t,x)^\dagger \Omega _\mu (t,x) \nonumber \\&\quad =\sum _{n=0}^\infty \frac{1}{(2n+1)!} \left( \frac{a}{2}D_\mu \right) ^{2n}\partial _t B_\mu (t,\tilde{x}), \end{aligned}$$

which explicitly contains even powers of *a* only. For the gradient force term in $F_\mu (t,x)$ we have only worked out the first few orders of the *a*-expansion explicitly. Therefore, the re-expansion cannot be carried out to all orders in *a* and it is thus advisable to resort to some more general argument based on symmetries.

### Reflection symmetries

We now consider the flow action in Eq. (41), but restricted to plaquette and rectangle terms, as this is sufficient to discuss the case of the Zeuthen flow. We consider a coordinate reflection ${\mathcal R}_\alpha$ in direction $\alpha$. The point with coordinates $x_\mu$ transforms into $x'_\mu$ with

126a $$\begin{aligned} {\mathcal R}_\alpha :\qquad x_\mu \longrightarrow x'_\mu = {\left\{ \begin{array}{ll} -x_\alpha , &{} \text { if }\mu = \alpha ,\\ x_\mu , &{} \text { if }\mu \ne \alpha , \end{array}\right. } \end{aligned}$$

The gauge field transforms under $\mathcal R_\alpha$

126b $$\begin{aligned} V_\mu (t,x) \longrightarrow {\left\{ \begin{array}{ll} V_\alpha (t,x' - a\hat{\alpha })^\dagger , &{} \text { if }\mu = \alpha ,\\ V_\mu (t,x'), &{} \text { if }\mu \ne \alpha , \end{array}\right. } \end{aligned}$$

One may then show that the gradient force terms (the RHS of the flow equation), transform for plaquette and rectangle terms, as follows:

126c $$\begin{aligned}&(\mu =\alpha ):\, X_\mu (t,x) \rightarrow \nonumber \\&\quad -V_\alpha (t,x'-a\hat{\alpha })^\dagger X_\alpha (t,x'-a\hat{\alpha }) V_\alpha (t,x'-a\hat{\alpha }) \nonumber \\&(\mu \ne \alpha ):\, X_\mu (t,x) \rightarrow X_\mu (t,x') \end{aligned}$$

In fact, the same transformation behaviour is found for the left hand side of the flow equation, so that Eq. (126c) equally holds for $F_\mu (t,x)$ of Eq. (10). Hence, if the same transformation behaviour (126c) is imposed on the Lagrange multiplier field, $L_\mu (t,x)$, we obtain for the different parts of the action density,

127 $$\begin{aligned} \begin{array}{ll} (\mu =\alpha ):\, &{}\mathrm{tr}(L_\mu (t,x) F_\mu (t,x)) \rightarrow \\ &{}\mathrm{tr}(L_\alpha (t,x-a\hat{\alpha }) F_\alpha (t,x'-a\hat{\alpha }))\\ (\mu \ne \alpha ):\, &{}\mathrm{tr}(L_\mu (t,x) F_\mu (t,x)) \rightarrow \\ &{}\mathrm{tr}(L_\mu (t,x') F_\mu (t,x')) \end{array} \end{aligned}$$

In particular, the action is invariant under such a reflection, as the only effect consists in a re-ordering of the terms in the sum over the $x_\alpha$-coordinate.^11^

### Example: reflection $\mathcal R_\alpha$ of the Wilson gradient force

It is instructive to derive Eq. (126c) for the case of the plaquette action in some detail. The gradient force in this case has the form

128 $$\begin{aligned} X_\mu (t,x) = \sum _\nu (P_{\mu \nu }(t,x) + Q_{\mu \nu }(t,x)^\dagger )_\mathrm{AH}, \end{aligned}$$

and we need to distinguish the two cases $\mu =\alpha$ and $\mu \ne \alpha$. Starting with $\mu =\alpha$ and setting $y = x'- a \hat{\alpha }$ we obtain the transformation behaviour of these plaquettes:

129a $$\begin{aligned} P_{\alpha \nu }(t,x) \longrightarrow V_\alpha (t,y)^\dagger P_{\alpha \nu }(t,y)^\dagger V_\alpha (t,y), \end{aligned}$$

and, similarly,

129b $$\begin{aligned} Q_{\alpha \nu }(t,x)^\dagger \longrightarrow V_\alpha (t,y)^\dagger Q_{\alpha \nu }(t,y) V_\alpha (t,y). \end{aligned}$$

Summing both expressions and taking the antihermitian part we thus obtain

130 $$\begin{aligned} \begin{array}{ll} &{}\left( P_{\alpha \nu }\right. (t,x) + \left. Q_{\alpha \nu }(t,x)^\dagger \right) _\mathrm{AH} \longrightarrow \\ &{}\quad - V_\alpha (t,y)^\dagger ( P_{\alpha \nu }(t,y) + Q_{\alpha \nu }(t,y)^\dagger )_\mathrm{AH} V_\alpha (t,y),\quad \end{array} \end{aligned}$$

where we have used the relation $(M^\dagger )_\mathrm{AH} = -(M)_\mathrm{AH}$, valid for any square matrix *M*.

Next we consider the case $\mu \ne \alpha$. The transformations of the plaquettes in this case read

131a $$\begin{aligned} P_{\mu \nu } (t,x) \longrightarrow {\left\{ \begin{array}{ll} Q_{\mu \alpha }(t,x')^\dagger , &{} \text { if }\nu = \alpha , \\ P_{\mu \nu }(t,x'), &{} \text { if }\nu \ne \alpha , \end{array}\right. } \end{aligned}$$

and

131b $$\begin{aligned} Q_{\mu \nu } (t,x)^\dagger \longrightarrow {\left\{ \begin{array}{ll} P_{\mu \alpha }(t,x'), &{} \text { if }\nu = \alpha ,\\ Q_{\mu \nu }(t,x')^\dagger , &{} \text { if }\nu \ne \alpha . \end{array}\right. } \end{aligned}$$

Hence, Eq. (126c) follows and the part of the lattice flow action containing the Wilson gradient force is indeed invariant under a reflection ${\mathcal R}_\alpha$. We have also verified that this remains true for any gradient force obtained from lattice actions containing both plaquettes and rectangles, such as the Lüscher–Weisz action.

### Lattice vs. continuum reflections

We now consider a total reflection, $\mathcal R = \mathcal R_0 \mathcal R_1 \mathcal R_2 \mathcal R_3$ of all space-time coordinates, i.e.

132 $$\begin{aligned} \mathcal R: \quad x \longrightarrow x' = -x \end{aligned}$$

The part of the flow action density for fixed index $\mu$ then transforms as follows:

133 $$\begin{aligned} \mathrm{tr}\{L_\mu (t,x) F_\mu (t,x)\} \longrightarrow \mathrm{tr}\{L_\mu (t,x'-a\hat{\mu }) F_\mu (t,x'-a\hat{\mu })\}. \end{aligned}$$

It is not difficult to see that the *O*(*a*) offset in this transformation is again an artefact of the asymmetric treatment of the links. In fact, defining again the midpoint $\tilde{x} = x+ \frac{1}{2} a\hat{\mu }$, and using the transformation of the “half link variables”, $\Omega (t,x)$ (122),

134 $$\begin{aligned} \mathcal R: \qquad \Omega _\mu (t,x) \longrightarrow \Omega _\mu \left( t,x'-\tfrac{a}{2}\hat{\mu }\right) ^\dagger , \end{aligned}$$

we find that the transformation behaviour of the fields at the midpoint is given by

135 $$\begin{aligned} \tilde{L}_\mu (t,\tilde{x})\longrightarrow & {} -\tilde{L}_\mu (t,-\tilde{x}), \end{aligned}$$

136 $$\begin{aligned} \tilde{F}_\mu (t,\tilde{x})\longrightarrow & {} -\tilde{F}_\mu (t,-\tilde{x}), \end{aligned}$$

i.e. the reflection $\mathcal R$, once expressed in terms of the fields at the midpoint $\tilde{x}$ takes the same form as its continuum counterpart. Therefore, the *a*-expansion of the corresponding part of the lattice flow action cannot generate terms that are odd under $\mathcal R$. A generic term transforms as follows:

137 $$\begin{aligned} T_{\mu _1\mu _2\ldots \mu _n}(t,\tilde{x}) \longrightarrow (-1)^n T_{\mu _1\mu _2\ldots \mu _n}(t,-\tilde{x}), \end{aligned}$$

so that terms with an odd number *n* of Lorentz indices can be excluded. This together with the observation that all Lorentz vectors ($D_\mu$, $L_\mu$, $\partial _t L_\mu$,...) have odd canonical dimension, implies that any term containing an even number of them must be even dimensional and thus be accompanied by an even power of *a*.

Treating the parts of the lattice flow action density with other values of $\mu$ in the same way, no odd powers of *a* can be generated in the expansions about the respective midpoints of the links relating $x+a\hat{\mu }$ and *x*. As these midpoints coalesce to a single point in the continuum limit this establishes this property for the *a*-expansion of the complete lattice flow action, for gradient force terms containing plaquette and rectangle terms.

For these considerations to extend to the Zeuthen flow we only need to check that the correction term,

138 $$\begin{aligned} \nabla _\mu ^*\nabla _\mu ^{} X_\mu (t,x), \end{aligned}$$

transforms like $X_\mu (t,x)$ itself under the reflection $\mathcal R$. This is indeed the case, so that the absence of odd powers of *a* is confirmed for the Zeuthen flow, too. Finally, while it is plausible that these considerations extend to gradient force terms derived from lattice gauge actions containing the “chairs” and “parallelograms”, we did not check this explicitly, as it is not needed for the discussion of the $O(a^2)$ improved Zeuthen flow.

## Action and heat kernels to $\mathcal O(a^2)$

### Free lattice actions and their kernels

The choice of observable, action and flow at tree level can be parameterised by the kernels of free lattice actions, i.e. the gauge action expanded to second order in the gluon fields, possibly supplemented by a gauge fixing term. If a generic lattice action with Wilson loops of length 4 and 6 is chosen then these are parameterised by a set of coefficients $c_i$, $i=0,1,2,3$. An alternative is provided by directly inserting the clover leaf definition of the gluon field strength tensor into a continuum like action density. In momentum space any of these actions is written

139 $$\begin{aligned} S_g[U] = \frac{1}{2} \int _p A_\mu ^a(-p) K_{\mu \nu }(p;\lambda ) A_\nu ^a(p) + \mathcal O(g_0). \end{aligned}$$

Note that for the case of a finite volume with twisted boundary conditions, the expressions of the kernels $K_{\mu \nu }(p;\lambda )$ are unchanged, but the integrals over momenta have to be substituted by sums and the momentum has to be interpreted as the sum of the space and colour momenta (see Eq. (95) and the subsequent discussion). Gauge fixing is performed in any kernel by adding the usual gauge fixing term

140 $$\begin{aligned} K_{\mu \nu }(p;\lambda ) = K_{\mu \nu }(p;0) + \lambda \hat{p}_\mu \hat{p}_\nu . \end{aligned}$$

Expanding the kernels to $\mathcal O(a^2)$ around their common continuum limit,

141 $$\begin{aligned} K(p;\lambda ) = K^\mathrm{cont}(p;\lambda ) + a^2 R(p,\lambda )+\mathcal {O}(a^4), \end{aligned}$$

with

142 $$\begin{aligned} K^\mathrm{cont}_{\mu \nu }(p;\lambda ) = p^2 \delta _{\mu \nu } +(\lambda -1) p_\mu p_\nu , \end{aligned}$$

the leading cutoff effects are encoded in the structure of $R_{\mu \nu }(p;\lambda )$. For example for a generic action made of an arbitrary linear combination of loops of four and six links Eq. (6) we have

143 $$\begin{aligned} \begin{array}{ll} K^{(G)}_{\mu \nu }(p;\lambda ) &{}= \hat{p}^2\delta _{\mu \nu } - (1-\lambda )\hat{p}_\mu \hat{p}_\nu \\ &{}\quad - a^2(c_1-c_2-c_3)[ (\hat{p}^4 + \hat{p}^2\hat{p}_\mu ^2)\delta _{\mu \nu } \\ &{}\quad - \hat{p}_\mu \hat{p}_\nu (\hat{p}_\mu ^2+\hat{p}_\nu ^2) ], \\ \end{array} \end{aligned}$$

and

144 $$\begin{aligned} \begin{array}{ll} R^{(G)}_{\mu \nu }&{}(p;\lambda ) = -\Bigg [ \left( \frac{1}{12}+c_1-c_2-c_3\right) p^4 \\ &{}+ (c_1-c_2-c_3)p^2p_\mu ^2 \\ &{}+(c_2+c_3)(p^2)^2\Bigg ]\delta _{\mu \nu }\\ &{}+ p_\mu p_\nu \Bigg [\left( \frac{1-\lambda }{24} +c_1-c_2-c_3\right) (p_\mu ^2+p_\nu ^2) \\ &{}+ (c_2+c_3)p^2 \Bigg ]. \end{array} \end{aligned}$$

The expressions for $K_{\mu \nu }$ and $R_{\mu \nu }$ for the other common choices of discretisations are written in Table 2. Note that the Zeuthen flow equation, even if it is not derived from the gradient of an action, can also be parametrised to tree level by a kernel. The main difference is that the property

145 $$\begin{aligned} K_{\mu \nu }(p;\lambda ) = K_{\nu \mu }(p;\lambda ), \end{aligned}$$

which is obeyed by any kernel derived from the gradient of an action (i.e. a consequence of the action being real), does not hold for the Zeuthen flow.

**Table 2:** Kernels $K_{\mu \nu }$ corresponding to different choices of discretisation, and discretisation effect corrections $R_{\mu \nu }$ for some of the most popular choices. See Appendix A for any unexplained notation

Discretisation	$K_{\mu \nu }(p;\lambda )$
Plaquette	$\hat{p}^2\delta _{\mu \nu } -(1-\lambda )\hat{p}_\mu \hat{p}_\nu$
Lüscher–Weisz	$\hat{p}^2\delta _{\mu \nu } -(1-\lambda )\hat{p}_\mu \hat{p}_\nu + \frac{a^2}{12}[ (\hat{p}^4 + \hat{p}^2\hat{p}_\mu ^2)\delta _{\mu \nu } - \hat{p}_\mu \hat{p}_\nu (\hat{p}_\mu ^2 + \hat{p}_\nu ^2)]$
Clover	$\mathring{p}^2\hat{c}_\mu ^2\delta _{\mu \nu } - \mathring{p}_\mu \hat{c}_\mu \mathring{p}_\nu \hat{c}_\nu$
Zeuthen	$(1-a^2\hat{p}_\mu ^2/12)\left\{ \hat{p}^2\delta _{\mu \nu } - \hat{p}_\mu \hat{p}_\nu + \frac{a^2}{12}[ (\hat{p}^4 + \hat{p}^2\hat{p}_\mu ^2)\delta _{\mu \nu } - \hat{p}_\mu \hat{p}_\nu (\hat{p}_\mu ^2 + \hat{p}_\nu ^2)]\right\} + \lambda \hat{p}_\mu \hat{p}_\nu$

Discretisation	$R_{\mu \nu }(p;\lambda )$
Plaquette	$- \frac{1}{12}\left[ p^4\delta _{\mu \nu } - (1-\lambda )\frac{1}{2}p_\mu p_\nu (p_\mu ^2 + p_\nu ^2)\right]$
Lüscher–Weisz	$\frac{1}{12}p^2p_\mu ^2\delta _{\mu \nu } - \frac{1+\lambda }{24}p_\mu p_\nu (p_\mu ^2+p_\nu ^2)$
Clover	$-\left( \frac{1}{3}p^4 + \frac{1}{4}p^2p_\mu ^2\right) \delta _{\mu \nu } + \frac{7}{24}p_\mu p_\nu (p_\mu ^2+p_\nu ^2)$
Zeuthen	$\frac{1}{24}p_\mu p_\nu [ (1+\lambda )p_\mu ^2 - (1-\lambda )p_\nu ^2 ]$

### Heat kernels and propagators to $\mathcal O(a^2)$

Given a kernel *K* with arbitrary value of the gauge parameter $\lambda$ (or $\alpha$ in the case of the flow kernel), we would like to obtain the $a^2$ correction term to either the propagator, i.e. the inverse of *K*,

146 $$\begin{aligned} D(p;\lambda ) = K(p;\lambda )^{-1}, \end{aligned}$$

or the heat kernel

147 $$\begin{aligned} H(t,p;\alpha ) = \exp \left( -t K(p;\alpha )\right) , \end{aligned}$$

given the expansion of the kernel Eq. (141).

Starting with the propagator, we can formally invert,

148

and then expand in $a^2$ to obtain

$$\begin{aligned} \begin{array}{ll} D(p;\lambda ) = &{}D^\mathrm{cont}(p;\lambda ) -a^2 D^\mathrm{cont}(p;\lambda ) R(p;\lambda ) D^\mathrm{cont}(p;\lambda ) \\ &{}+\, \mathcal O(a^4). \end{array} \end{aligned}$$

To work out the $\mathcal O(a^2)$ piece of the heat kernel we define the transverse and longitudinal projectors

149 $$\begin{aligned} T_{\mu \nu }(p) = \delta _{\mu \nu } - \frac{p_\mu p_\nu }{p^2},\quad L_{\mu \nu }(p) = \frac{p_\mu p_\nu }{p^2}, \end{aligned}$$

in terms of which

150 $$\begin{aligned} K^\mathrm{cont}_{\mu \nu }(p;\alpha ) = p^2 [T_{\mu \nu }(p) + \alpha L_{\mu \nu }(p) ], \end{aligned}$$

and the heat kernel in the continuum is given by

$$\begin{aligned} H^\mathrm{cont}(t,p;\alpha ) = \mathrm{e}^{-t K^\mathrm{cont}(p;\alpha )} = \mathrm{e}^{-tp^2}T(p) + \mathrm{e}^{-\alpha tp^2} L(p). \end{aligned}$$

Note that $K^\mathrm{cont}(p;\alpha )$ and $R(p;\alpha )$ do in general not commute. Nevertheless, it is not difficult to work out the expansion to $\mathcal O(a^2)$ (i.e. to first order in $R(p;\alpha )$). Inserting (141) in the exponent one obtains

151 $$\begin{aligned} \mathrm{e}^{-tK(p;\alpha )} = \mathrm{e}^{-tp^2} \mathrm{e}^{u L(p) + v R(p;\alpha )} + \mathcal {O}(v^2), \end{aligned}$$

with

152 $$\begin{aligned} u=(1-\alpha )p^2 t,\quad v=-a^2 t . \end{aligned}$$

Then noting that, for $n>1$,

$$\begin{aligned} (uL +vR)^n= & {} u^n L + u^{n-1} v \{LR+(n-2)LRL +RL\} \\&+\mathcal {O}(v^2), \end{aligned}$$

the exponential series can be resummed with the result

$$\begin{aligned} \begin{array}{ll} \mathrm{e}^{u L + v R} &{}= \sum _{n=0}^\infty \frac{(uL+vR)^n}{n!} \\ &{}= T + \mathrm{e}^u L+v\left[ R-\overline{R} + \frac{\mathrm{e}^u-1}{u}(\bar{R} + u LRL)\right] \\ &{}\quad +\, \mathcal {O}(v^2), \end{array} \end{aligned}$$

where

153 $$\begin{aligned} \bar{R} = LR + RL -2LRL. \end{aligned}$$

The result for the heat kernel then is

154 $$\begin{aligned} H(t,p;\alpha )= & {} H^\mathrm{cont}(t,p,\alpha ) + a^2 t \mathrm{e}^{-tp^2}\biggl \{\bar{R} -R \nonumber \\&\, +\,\frac{1-\mathrm{e}^{(1-\alpha )tp^2}}{(1-\alpha )tp^2} [\bar{R} + (1-\alpha )tp^2 LRL]\biggr \}\nonumber \\&+ \mathcal {O}(a^4), \end{aligned}$$

where we have left out the arguments for the sake of readability. Note that the choice of Feynman gauge $\alpha =1$ for the heat kernel is not a problem, as the apparent singularity cancels, with the result

155 $$\begin{aligned} H(t,p,;1)= \mathrm{e}^{-tp^2}\{1-a^2 t R(p;1) \} + \mathcal {O}(a^4). \end{aligned}$$

## Footnotes

1 The radius $r_t=\sqrt{8t}$ amounts to two standard deviations in the Gaussian smearing function which appears in the relation between $B_\mu (t,x)$ and $A_\mu (x)$ to leading order in the coupling.

2 Note, however, that the choice of the coefficients is not completely free, with some constraints arising from positivity [19]. Our conventions differ from this reference by the exchange $c_2 \leftrightarrow c_3$.

3 For a more careful discussion of the limits involved cf. [4].

4 We assume here that the quark fields only live at $t=0$, i.e. they are not propagated into the $4+1$-dimensional bulk. For generalisations cf. [4].

5 Such boundary conditions are often imposed in the Euclidean time direction, combined with periodic boundary conditions in the spatial directions. In this case one may distinguish between the electric and magnetic components of *E*(*t*, *x*). In the latter, total derivatives only appear in the spatial directions and thus do not contribute to the expectation value.

6 While the continuum derivation may seem rather formal we note that a lattice version of this equation can be derived directly from the $\varepsilon$-regularised $4+1$-dimensional lattice action by a variation with respect to the link field $U_\mu (x)$, followed by the limit $\varepsilon \rightarrow 0$.

7 Note that all the improvement coefficients $c_i$ and $c_b$ have a perturbative expansion of the form $c(g_0^2) = c^{(0)} + g_0^2c^{(1)} + \cdots$. Since we are only concerned with tree-level improvement we will omit the superscript ${}^{(0)}$ in all improvement coefficients.

8 See Appendix A for a summary of our notation and conventions.

9 Note that higher orders in the fields imply higher order in the coupling, too, cf. Eq. (69).

10 Negative powers ($j<0$) have to be understood as in the infinite volume, Eq. (89).
